# Supplementary material for: Digital imaging and vision analysis in science project improves the self-efficacy and skill of undergraduate students in computational work
Source: PLoS One. 2021 May 5;16(5):e0241946. doi: 10.1371/journal.pone.0241946 (PMC8099079; doi:10.1371/journal.pone.0241946)
Supplement: S1 File — (PDF) [file pone.0241946.s001.pdf]

DIVAS Seminar I
BIO 271
1 credit

Class hours: Thursday, 3:00-3:50 PM in Lied 131

Resources:

- Course materials and resource are available on Slack in the #seminar_i_discussion.
- Instead of email, use Slack for any class-related communication. This will make our communication more efficient. You can download the Slack app to your phone and use it to ask questions, communicate with each other and DIVAS faculty, and complete occasional assignments.

Course Description

This seminar is an introduction to the Doane Digital Imaging and Vision Applications in Science (DIVAS) Project. Students will learn about the many areas of the natural sciences where image data is used including medicine, agriculture, and analytical chemistry. Students will learn about the technologies used to collect image data and will learn what makes a good image. Students will meet professionals who work with images and will be introduced to the collaborative work environment they utilize in their projects. Students who have been selected as DIVAS scholars are required to enroll in this seminar before starting summer research. Note: This course is part of an ongoing research study, therefore any products of the course will be incorporated into that research.

Goals & Objectives

1. Describe what an image is, how it's made up, and the technologies used to collect them.
2. Be able to assess what makes an image useful for data collection and computing applications.
3. Increase your awareness of how image data and coding is used in professional settings.
4. Start to answer your own questions using image capture and analysis.
5. Investigate how image data and coding might be used in your future career environment.
6. Utilize communication methods and tools common to professional settings.

Assessments (all point totals are approximate)

- **Weekly Reflections and Discussion**
  Each week, you will be given a short reading or video assignment. You will post responses to those readings before class each week.
- **Photo Diary**
  You will take and compile an image collection using your smartphone or other camera centered around something you are interested in (e.g. how long does it take a Twinkie to mold, who's in the LC, the salad bar composition, a tree or tree limb over semester, squirrels on campus, the view from your favorite place on campus, your wardrobe each day, etc etc.). You will examine your diary as we learn more about what an image is composed of and what makes an image useful for research. You will present an analysis of your diary at the end of the semester.
- **Professional Explorations**
  We will meet professionals virtually or person-to-person through three Professional Explorations. For each exploration, we will do some background research on the person we are meeting, will conduct a virtual or

in-person tour of their work environment, and will follow up with discussion, research, and follow-up questions.

- **Mind Expo Presentation**
  You will summarize your photo diaries, analysis, and DIVAS experience in a presentation at Mind Expo. All students are required to participate in preparing the presentation and giving the presentation. Please begin talking to your professors and coaches early in the semester so that you can be present at MindExpo!

All assignments must be handed in by the due date (see Attendance and Make-up Work below). Late assignments will lose 30% of the points possible. Any assignment turned in for an unexcused absence will receive 0%.

Grading

Final grades will be determined by the following (all point totals are approximate):

Weekly reflections (including professional explorations)	110 pts
Photo diary and analysis	60 pts
MindExpo Presentation	40 pts
TOTAL	200 pts

Your final grade will be determined as a percentage of the total points possible.

Grading Scale

Grades will be based on the following scale:

A+ 97-100%
A 93-96%
A- 90-92%
B+ 87-89%
B 83-86%
B- 80-82%
C+ 77-79%
C 73-76%
C- 70-72%
D+ 67-69%
D 63-66%
D- 60-62%
F <59%

ADDITIONAL POLICIES

Attendance and Make-up Work
In order to excel in this course, your priority should be to attend every class. On occasion, everyone has some personal emergency. You will be responsible for deciding whether your personal emergency merits missing class. If in doubt as to whether your absence will be excused, ask! If you miss class for **ANY** reason, it is your responsibility to get the notes, handouts and assignments from a classmate or from me.

Excused make-up work: In order to excuse an absence it must be cleared with me at least three days in advance. To excuse unplanned absences (i.e. illness and/or emergency) you must contact me as soon as possible after missing class. Assignments that are excused must be turned in within one week of the absence to receive full credit.

Unexcused absences: Work missed as a result of an unexcused absence receives no credit.

Academic Integrity
Plagiarism and cheating are unacceptable in this class and in your profession. Academic dishonesty includes, but is not limited to, cheating, fabrication, helping or attempting to help someone else commit dishonesty (facilitating), and plagiarism. These policies are more fully discussed in your Student Handbook. **As faculty, we are required to report every instance of academic dishonesty.** If you are guilty of academic dishonesty, possible outcomes include: being asked to resubmit an assignment, receiving a reduced grade or a grade of F on the assignment, receiving a reduced grade for the course, or receiving a grade of F for the course.

UNIVERSITY POLICIES AND SUPPORT

Technical Support Contact Information

If you are in need of technical assistance please access the Self Service Portal. You may reach the help desk at 402-826-8411 or by email at helpdesk@doane.edu.

Academic Integrity

Fundamental to our mission, our core values, and our reputation, Doane University adheres to high academic standards. Students of Doane University are expected to conduct themselves in a manner reflecting personal and professional integrity. Disciplinary actions may be taken against students whose academic behavior is not congruent with the expectations of the University. Students are responsible for adhering to the standards detailed in this policy. Not being familiar with these standards does not mean that the students will not be accountable for adherence to them. Additional details on the Academic Integrity policy for violating academic integrity are published in the undergraduate and graduate catalogs.

Accommodations

Any student who thinks they may need an accommodation based upon the impact of a disability should contact the Office of Disability Services (https://www.doane.edu/disability-services) to coordinate reasonable accommodations as soon as possible.

Course Participation

Doane University expects active participation by a student in a course, whether the course is on-ground or online. A student is expected to be prompt and regularly attend on-ground classes in their entirety.

Syllabus Changes

The instructor and Doane University reserve the right to make changes as necessary to this course syllabus. All students will be notified of any changes.

Doane Syllabus Addendum

Each student is responsible for being aware of the policies, resources, and expectations as specified in the Doane Syllabus Addendum found at: https://www.doane.edu/Syllabus

Mandatory Reporter Statement

At Doane, all university employees, including faculty, are considered Mandatory Reporters. As a“Mandatory Reporter, I am required to report incidents of sexual misconduct and relationship violence to the Title IX Coordinator and, thus, cannot guarantee confidentiality. This means that if you tell me about an incident of sexual harassment, sexual assault, domestic violence, dating violence, stalking and/or other forms of prohibited discrimination, I have to share the information with the University’s Title IX Coordinator. My report does not mean that you are officially reporting the incident. This process is in place to ensure you have access to and are able to receive the support and resources you need. Please learn additional information (including confidential resources) at doane.edu/cape-project

Tentative Schedule

Week	Topic	Assignments Due
1	Pre-assessments	Install Slack on computer and phone<br>Log onto ‘DIVAScholars’’ team
2	Meet with DIVAS II Seminar	Bring questions for DIVA Scholars<br>Prompt 1 in #seminar_i_discussion
3	What are images used for? Tour of Doane image data	Images as data exploration<br>Prompt 2 and response in #seminar_i_discussion
4	What is an image?<br>Finish tour of Doane image data	ImageJ and Image tutorial video (up to 19:00)
5	Photo diary project overview<br><br>Overview of Professional Explorations	Prompt 3 and response in #seminar_i_discussion
6	Photo diary ideas and project selection	Photo Diary Project selection and initial photo collection<br><br>Prompt 4 and response in #seminar_i_discussion<br>Follow-up #seminar_i_discussion
7	NO CLASS	Exploration 1 documents*<br><br>Photo Diary Week 1 Images
8	Professional Exploration 1<br><br>ImageJ analysis of photo diary images	Photo Diary Prompt #1 in #seminar_i_discussion<br>Photo Diary Week 2 Images<br><br>Mind Expo registration due March 8
	**SPRING BREAK**	
9	Catching up	Photo Diary Prompt #2 and response in #seminar_i_discussion
10	ImageJ debrief and discuss analysis ideas, HUDL exploration	Photo Diary Prompt #3 and response in #seminar_i_discussion<br><br>Photo Diary Week 3 Images
11	Finish image analysis<br><br>HUDL virtual visit	Questions for Casey Bateman, HUDL<br><br>Photo Diary Week 4 Images
12	Photo Diary presentations, Intro to Python Programming	Photo Diary presentation
13	Rosalind/Python debrief	Rosalind Exercise
14	Rosalind/Python debrief	Rosalind Exercise
Finals	**Summer Preview and Planning**<br>**Post-assessments**	**11:30-2:30 PM**
