# Supplementary material for: Digital imaging and vision analysis in science project improves the self-efficacy and skill of undergraduate students in computational work
Source: PLoS One. 2021 May 5;16(5):e0241946. doi: 10.1371/journal.pone.0241946 (PMC8099079; doi:10.1371/journal.pone.0241946)
Supplement: S2 File — (PDF) [file pone.0241946.s002.pdf]

Photo Diary Assignment

Similar project in the Graphic Design field

Description: In this project, you will come up with an idea for an image collection. Your collection should be completed over four weeks. You will collect at least four images per week. You should collect at least sixteen images on sixteen separate days.

Each week, you will be asked to reply to a prompt. Your reflections will include your observations (what's interesting about the images you've collected this week?), any struggles you've encountered, and the adjustments you want to make for the next week.

At the end of the semester, you will reflect on what your images mean to you as a whole. You'll also step back from them to think about how they could be viewed as a source of data and will start doing some analysis using ImageJ after spring break. Your photo diary project will be presented at MindExpo.

Instructions: In the space below, write a short description of your photo diary. Include information on what images you plan to take, how you will take them (using what kind of camera) and how often they will be captured. Store your images in the 'Photo Diary 2020' folder shared with you in Google Drive. You can view these instructions and the diaries of your colleagues in this folder as well. Occasionally, you will be given prompts to reply to on Slack reflecting on your photo diary.

> Student 1: My photo diary will be focused on the changing of seasons based on trees. I will be using a Canon Rebel T6 to take images of different trees around campus. I will take close up photos of the branches/leaves. I will find two different trees to image and take pictures of them at least twice a week. I am hopeful that with spring approaching, the branches will start to grow new leaves soon.
>
> Student 2: In my photo diary, I will be observing a blackberry rotting. I will be using my iphone to take crisp photos of the rotting/decomposition process. I intend to grab one photo (Monday-Friday) for four weeks.
>
> Student 3: For my photo diary assignment I will be taking photos of a sapling outside Sheldon Hall. It is in sunlight for most of the day. I'd like to take pictures of the ends of one of the branches to see how it develops as we (hopefully) transition into warmer weather. I will be taking photos with my Samsung Galaxy S9 camera. I will take pictures close to noon 4 times a week for the duration of the project.
>
> Student 4: for my photo diary I will be recording the coffee shop refrigerator and note what items get selected and at what rate, I just attempt to record once a day or more and usually around closing time. Closing time is at 10pm. I want to see the behavior in which the students behave on campus and see their snack/drink habits. I will be taking photos with an iPhone XS.

(See Weekly Discussion Prompts Examples for follow up assignments and Syllabus for image analysis and presentation scheduling)
