# Supplementary material for: Digital imaging and vision analysis in science project improves the self-efficacy and skill of undergraduate students in computational work
Source: PLoS One. 2021 May 5;16(5):e0241946. doi: 10.1371/journal.pone.0241946 (PMC8099079; doi:10.1371/journal.pone.0241946)
Supplement: S3 File — (PDF) [file pone.0241946.s003.pdf]

Prompt 1

In this post, talk about what drew you to applying for the DIVA Scholars program and what you are looking forward to this semester and summer. Next week, last year's Scholars will be coming to class. What questions do you have for them? Post your reflection and questions in preparation for their visit.

Prompt 2

Search the internet for examples of images that are used as data. You can search any area in the sciences, design, engineering, architecture, archeology, etc. A couple sites you could start in (that are biologically related) are iDigBio (https://www.idigbio.org) and the Visible Human (https://www.nlm.nih.gov/research/visible/getting_data.html). The UNL Center for Digital Research in the Humanities (https://cdrh.unl.edu/) also has some fascinating examples of non-life sciences image data to start you off, or you might consider looking at museum collections, collections of maps, etc. **MAKE SURE YOU FIND IMAGES USED AS DATA (not an image of some other form or data or an image of something that is not data).** Try to find a collection or libraries of images in an area you are particularly interested in or that just "speaks to you".

After doing your search, select 2-3 of the images you find and post them to #seminar_discussion. For each image, provide:

- a short description telling us what it is
- the magnification of the image (if known)
- Information on how/when/where the image was taken (as far as you can determine)
- A short statement of what you see as the most interesting or useful feature or aspect of each image.

Prompt 2 Response

Make comments on the posts/images for at least two other students. You can react or add replies to additional posts as desired. In your comments, talk about what interests you about the images chosen and how you think they could be used (for example, What might you try to measure or quantify?, Would you use one image by itself or compare to other images?). Also include any additional information you would like to have about the images to understand how they are used as data.

Prompt 3

Do some internet browsing to find example of photo diaries. Here's one on Cats of Greece and another of a person who shaved their hair and imaged it every day for a year. The diary could be done at any scale about any topic. In other words, anything

from the scale of microscopy to telescopy and from the mundane to the insane could be explored. Once you have found at least one other example, post it and respond to the following:

- Describe the diary. Who compiled it? For what purpose?
- How was the imaging done? What can you gather about the type of equipment used and how images were collected? Where images modified before compiling into the diary?
- What ideas do you have about your own diary?

Prompt 3 Response

Make comments on the posts for at least two other students. You can react or add replies to additional posts as desired. In your comments, address both the photo diary found and the ideas the student submitted for making their own photo diary.

Photo Diary Post #1

In this post, reflect on the images you've collected so far.

What has struck you as interesting about your image collection or the collection process this last two weeks?

This might include unforeseen issues in the collection process, any improvements or adjustments to that process, initial observations of variables, including unexpected or anticipated patterns, anything learned about the composition of an image, etc. This is very open ended. You are encouraged to express anything you think is pertinent to the experiment and the discovery process.

This response should cover the work between February 20th-March 5 (8 images should be complete).

Photo Diary Post #2

In this post, first reflect on your transition to remote/online learning.

Write a paragraph or two describing how you're adjusting to the change in format within your academics. Consider things like the change in your physical location, the way you are engaging with your instructors and classmates, the change in the routine of the course and the change in schedule. Also consider changes in your own routine that are happening and how you see that impacting your interactions with this course. What are you feeling good about? What concerns do you have? What are you unsure about?

Finally, reflect on your ideas for transitioning your photo diary project. Do you want to adapt your original idea to your current surroundings? Do you want to start something new that has grabbed your attention? Describe your new photo diary project and collect at least an image or two for us to look at next week. Post one of the photos of your new project in your post.

Photo Diary Post #2 Response

Make comments on the posts for at least two other students. You can react or add replies to additional posts as desired. In your comments, address both the photo diary found and the ideas the student submitted for making their own photo diary.

Prompt 4 - Professional Exploration 1

Our guest next week will be X from Y. During our seminar on Tuesday you will have the chance to have a conversation with him about his science, image analysis, his career path, and anything else that interests you.

To prepare for that discussion, visit this website and explore projects this person is a part of:

www.examplesite.org

Additionally, explore the web for information on field X and field Y. The links below are a starting point; you should explore them and others:

- Link1
- Link2
- Link3

Use the links above to explore these topics, then answer the below:

- [Questions asking students to definitions of the field the person is involved in and how the work this person does contributes broadly]
- [Prompt asking students to share an interesting example of the type of work this person does]
- [Prompt asking students to share an interested example of the type of software a person in this field might use]
- [Prompt asking students to think about an application or problem relevant to the field the person visiting works within]

Prompt 4 Response - Professional Exploration 1

Based on your explorations above, and your classmates' explorations, suggest 3-5 topics that you would like to discuss with Person X when they visit. These can be broad or specific and can be anything you want: science, image analysis, career path, grad school or research advice…

For each topic,

- Explain why it interests you
- Suggest some specific questions you could ask Alex to find out more
- If someone has already suggested your topic, add your thoughts and questions onto that thread as a reply.

Our goal is to develop a list of questions/topics to make sure that you get as much as you can from this visit!

Prompt 5 - Professional Exploration 2

Next week, we will be taking a field trip to Lincoln to visit a local software company, Hudl.

To prepare for that discussion, visit their website and explore the products that they offer:

Main webpage: https://www.hudl.com/

Jobs at Hudl: https://www.hudl.com/jobs

Make sure that you dig into the different sections of their website!

Then answer the questions below:

- Summarize what Hudl's business is about. What products/services do they offer?
- What do you think is the most interesting/exciting thing that they do, and why?
- What kinds of computer skills do you think working at Hudl requires? What role does image analysis and/or computer vision play in their work?
- Based on the images/descriptions on their website, what do you think their working environment is like? How is it different from a traditional office setting?

Prompt 5 Response - Professional Exploration 2

Based on your explorations above, and your classmates' explorations, suggest 2-3 topics that you would like to learn more about during our visit. These can be broad or

specific and can be anything you want: science, image analysis, career path, grad school or research advice…

For each topic,

- Explain why it interests you
- Suggest some specific questions you could ask to find out more
- If someone has already suggested your topic, add your thoughts and questions onto that thread as a reply.

Plan to bring your list of questions/topics with you to make sure that you get as much as possible from this field trip!
