# Supplementary material for: Digital imaging and vision analysis in science project improves the self-efficacy and skill of undergraduate students in computational work
Source: PLoS One. 2021 May 5;16(5):e0241946. doi: 10.1371/journal.pone.0241946 (PMC8099079; doi:10.1371/journal.pone.0241946)
Supplement: S4 File — (PDF) [file pone.0241946.s004.pdf]

# BIO 371 DIVAS Seminar II

## Spring 2020 Syllabus

### General Information

| | |
|---|---|
| Professor | Mark M. Meysenburg |
| Office | LI 231 |
| Phone (Office) | 402-826-8267 |
| Phone (Cell) | 402-418-1057 |
| E-mail | mark.meysenburg@doane.edu |
| Office hours | MTWRF 8:00 - 9:00 a.m., 10:00 - 11:00 a.m. |
| Class meetings | R, 3:00 - 3:50 in LI 238 / 131 |
| Prerequisites | BIO 271 DIVAS Seminar I |
| Credits | 1 |
| Text | - No text required |

### Course Description

This seminar is a capstone to the Doane Digital Imaging and Vision Applications in Science Project and is intended for DIVA scholars. Students mentor incoming DIVA scholars by participating in DIVAS Seminar I. Students will also help prepare for summer coding workshops by providing feedback and assistance with code documentation and tutorial content. Students will clean up code developed as a DIVA scholar and will present a summary of their research to students in DIVAS Seminar I. Students will compile their activities associated with the project into a brief portfolio. Finally, students will investigate how to implement image processing code in Python using a computer vision library other than OpenCV. DIVA scholars are required to enroll in this course after completing summer research.

### Learning Outcomes

Doane University's mission is to provide an exceptional liberal arts education in a creative, inclusive, and collaborative community where faculty and staff work closely with undergraduate

and graduate students, preparing them for lives rooted in intellectual inquiry, ethical values, and a commitment to engage as leaders and responsible citizens in the world.

## Instructional Details

We will use a variety of teaching styles in this course, including lecture, class discussion, hands-on bash and git practice, and hands-on programming. Throughout, interaction between the students and the professor is desired. In all of these activities, you must be actively involved!

## Course Evaluation

We will participate in several activities during the semester: cleaning up and organizing the summer research code repository, cleaning up and commenting the code in the repository, preparing and presenting a MindExpo oral presentation, preparing and presenting a capstone portfolio, and reviewing / improving the latest version of the DIVAS image processing workshop.

### Specifications Grading

This course uses the “specifications grading” methodology. In a traditional course, everyone completes the same assignments and gets letter or percentage grades on each assignment, with the final course grade calculated based on total points earned or on some formula involving all the assignments, quizzes, tests, etc.

In a specifications grading system, on the other hand, all assignments are evaluated on a high-standards, pass / fail basis, using detailed checklists of assignment requirements and expectations. Letter grades are earned by the satisfactory completion of a certain number and kind of assignments. Students choose what letter grade they desire and complete all assignments designated as part of that letter grade.

This system allows you as a student much more choice, and is closer to the kind of real-world experiences that adult life entails -- especially life as a programmer. This system is more rigorous than traditional grading and builds on well-documented links between high expectations and student success. It creates a safe but challenging environment in which you will thrive, because each assignment is assessed pass / fail, and the requirements for a pass are always clearly delineated. It will be easy for you to tell whether your work is complete, done in good faith, and consistent with my expectations and the intended learning outcomes of the course.

As you will see below, it is up to you what final letter grade you earn in this course. Your satisfactory completion of activities will determine that grade. You might choose to wade into the course content and skills by fulfilling the C-level requirements. You might want to take things further and swim around by additionally completing the B-level requirements. Or, you might want to go the distance and dive right in with the A-level challenge. Regardless of your path, I’m here to help. The choice is yours!

The following tables lay out the requirements for each grade, first for your midterm grade and then for your final course grade.

### Midterm Grading

| To receive this midterm grade... | … you must satisfy all of these requirements |
|---|---|
| D | * Pass at least 60% of the activities before mid-term<br>* At most one unexcused absence |
| C | * Pass at least 70% of the activities before mid-term<br>* At most one unexcused absence |
| B | * Pass at least 80% of the activities before mid-term<br>* No unexcused absences |
| A | * Pass at least 90% of the activities before mid-term<br>* No unexcused absences |

### Final Grading

| To receive this final grade... | … you must satisfy all of these requirements |
|---|---|
| D | * Pass at least 60% of the activities in the course<br>* At most one unexcused absence |
| C | * Pass at least 70% of the activities in the course<br>* At most one unexcused absence |
| B | * Pass at least 80% of the activities in the course<br>* No unexcused absences |
| A | * Pass at least 90% of the activities in the course<br>* No unexcused absences |

## Tentative Schedule

### Thursday, January 16

DIVAS II course introduction in LI 238

### Thursday, January 23

Introduction to DIVAS I cohort in LI 131
Bash, git review and practice

Thursday, January 30

DIVAS summer research repo clean-up

Thursday, February 6

Python review and practice

Thursday, February 13

DIVAS summer research code clean-up

Thursday, February 20

Image processing with Scikit-Learn

Thursday, February 27

MindExpo abstract preparation

Thursday, March 5

MindExpo abstract preparation / registration

Thursday, March 12

No class - Spring Break

Thursday, March 19

Image processing with Scikit-Learn

Thursday, March 26

Image processing with Scikit-Learn

Thursday, April 2

No DIVAS II - attend DIVAS I in LI 231

Thursday, April 9

MindExpo presentation practice

Thursday, April 16

MindExpo presentation

Thursday, April 23

Portfolio development

### Thursday, April 30

Portfolio presentation

## Other Information

### Technical Support Contact Information

If you are in need of technical assistance please access the Self Service Portal. You may reach the help desk at 402-826-8411 or by email at helpdesk@doane.edu.

### Academic Integrity

Fundamental to our mission, our core values, and our reputation, Doane University adheres to high academic standards. Students of Doane University are expected to conduct themselves in a manner reflecting personal and professional integrity. Disciplinary actions may be taken against students whose academic behavior is not congruent with the expectations of the University. Students are responsible for adhering to the standards detailed in this policy. Not being familiar with these standards does not mean that the students will not be accountable for adherence to them. Additional details on the Academic Integrity policy for violating academic integrity are published in the undergraduate and graduate catalogs.

### Course-Specific Academic Integrity Items

The following penalties will be applied in this class if an instance of academic dishonesty occurs. For the first offense, plagiarism or other dishonesty on any of the graded class activities will result in a failure for the activity. The gift certificates will have no effect on an assignment failed for an academic honesty violation. A second occurrence of dishonesty will result in a grade of 'F' for the course.

You are expected to attend each class. If you must miss class for a test or presentation, notify the instructor BEFORE the class. If you do not notify the instructor before missing a test or presentation, you will not be allowed to make it up.

You will be programming in pairs this semester, so you must confer with your partner about your assignment. You may not confer with anyone else about the assignment. You should definitely not share your finished work with another pair, nor ask another pair to share their work with you. Do not do anyone else’s work for them, nor copy another student’s work, nor download all or part of an assignment from the Internet.

A simple rule of thumb will keep you safe: Never show your code to anyone except for your partner on the assignment, and do not look at someone else’s code. If you cannot orally explain your work to the instructor’s satisfaction, you will be considered to be in violation of the academic dishonesty policy.

You should also know that automated tools will be examining the code you submit for assignments, comparing it to the code of every other pair, to determine if plagiarism has occurred.

### Accommodations

Any student who thinks they may need an accommodation based upon the impact of a disability should contact the Office of Disability Services (https://www.doane.edu/disability-services) to coordinate reasonable accommodations as soon as possible.

### Course Participation

Doane University expects active participation by a student in a course, whether the course is on-ground or online. A student is expected to be prompt and regularly attend on-ground classes in their entirety. Regular engagement is expected for online courses.

You are expected to attend each class period, and to actively participate in the course discussions and activities involved.

### Syllabus Changes

The instructor and Doane University reserve the right to make changes as necessary to this course syllabus. All students will be notified of any changes.

### Doane Syllabus Addendum

Each student is responsible for being aware of the policies, resources, and expectations as specified in the Doane Syllabus Addendum found at: https://www.doane.edu/Syllabus
