# Supplementary material for: Digital imaging and vision analysis in science project improves the self-efficacy and skill of undergraduate students in computational work
Source: PLoS One. 2021 May 5;16(5):e0241946. doi: 10.1371/journal.pone.0241946 (PMC8099079; doi:10.1371/journal.pone.0241946)
Supplement: S5 File — (PDF) [file pone.0241946.s005.pdf]

bash / git review

Spring 2020

Common bash commands

See a resource like this

1. Listing directory contents: **`ls`**
2. Changing directory: **`cd`**
3. Making a directory: **`mkdir`**
4. Create empty file / update its timestamp: **`touch`**
5. List contents of a file to the screen: **`cat`** or **`less`**
6. Move a file: **`mv`**
7. Copy a file: **`cp`**
8. Remove a file: **`rm`**
9. Get help on a command: **`man`** or **`info`**
10. Editor: **`nano`**

git concept

git workflow

1. Check to see if you have any uncommitted work: **`git status`**
2. Get newest version of codebase: **`git pull origin master`**
3. Do your work; when you have reached a milestone,...
4. Stage your changes: **`git add -A`**
5. Add your changes to the local repo: **`git commit -m "<some message>"`**
6. Push your changes to the remote repo: **`git push origin master`**

If you need to clone the repo on a new machine: **`git clone <url>`**

Your tasks

Look through the DIVAS repo. Make sure the projects you worked on over the summer are listed in the `DIVAS projects.ods` spreadsheet (or provide updates in a text file if you can't edit the spreadsheet). Make sure the code for those projects is in the appropriate directories of the project folder.
