# Supplementary material for: Digital imaging and vision analysis in science project improves the self-efficacy and skill of undergraduate students in computational work
Source: PLoS One. 2021 May 5;16(5):e0241946. doi: 10.1371/journal.pone.0241946 (PMC8099079; doi:10.1371/journal.pone.0241946)
Supplement: S6 File — (PDF) [file pone.0241946.s006.pdf]

BIO 371 DIVAS II Seminar

Introduction to Parallelism

Outline

- Introduction to parallelism
  - Difference from sequential programs
  - Rationale for parallelism
  - Parallel computing architectures
  - Design of parallel programs
- Monte Carlo estimation of π
  - Three versions: sequential, OpenMP, OpenMPI

Warning!

This is only the slightest introduction to the field! Parallelism is complicated, still slightly immature field; could easily be a whole graduate class!

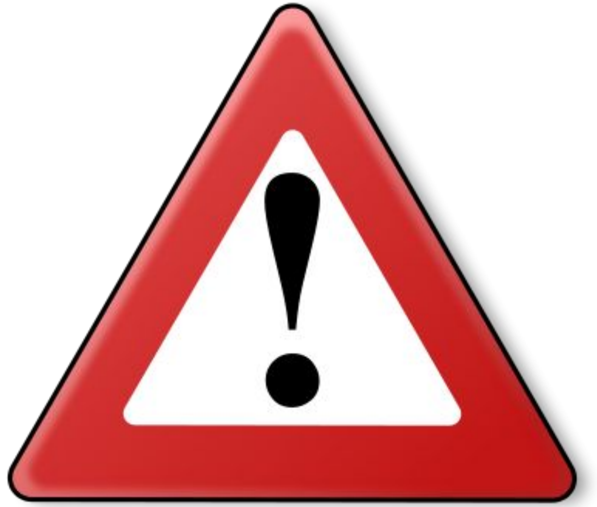

How are parallelized programs different from traditional programs?

Difference from sequential programs

- In sequential processing, the problem is broken down into steps, which are then solved in sequence on the CPU
- Only one step is "active" at any one time

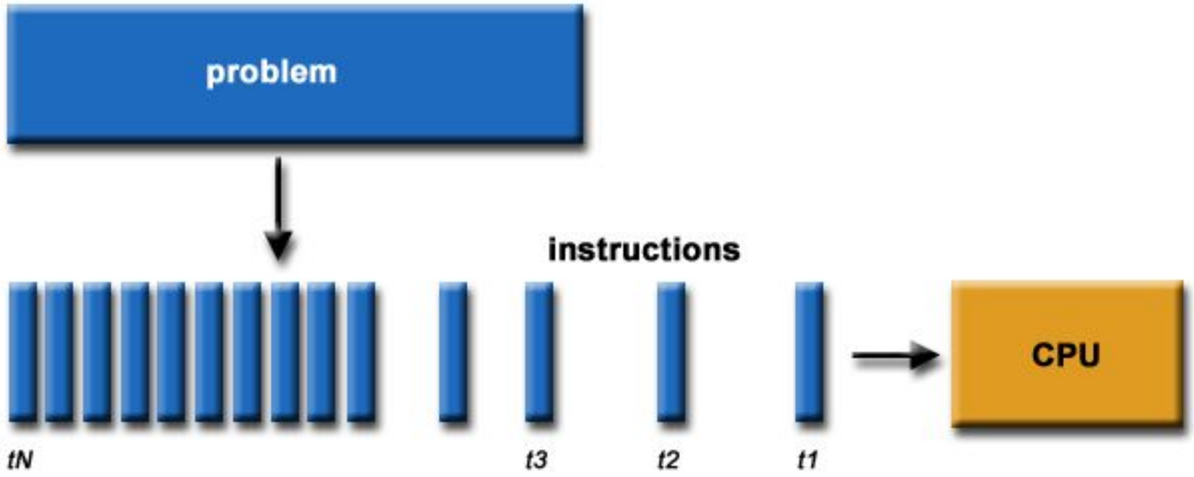

Traditional, uni-processor program

Difference from sequential programs

- In parallel computing, the problem is broken down into pieces that can run concurrently on multiple processors
- Each piece might be composed of several steps to be run on that processor

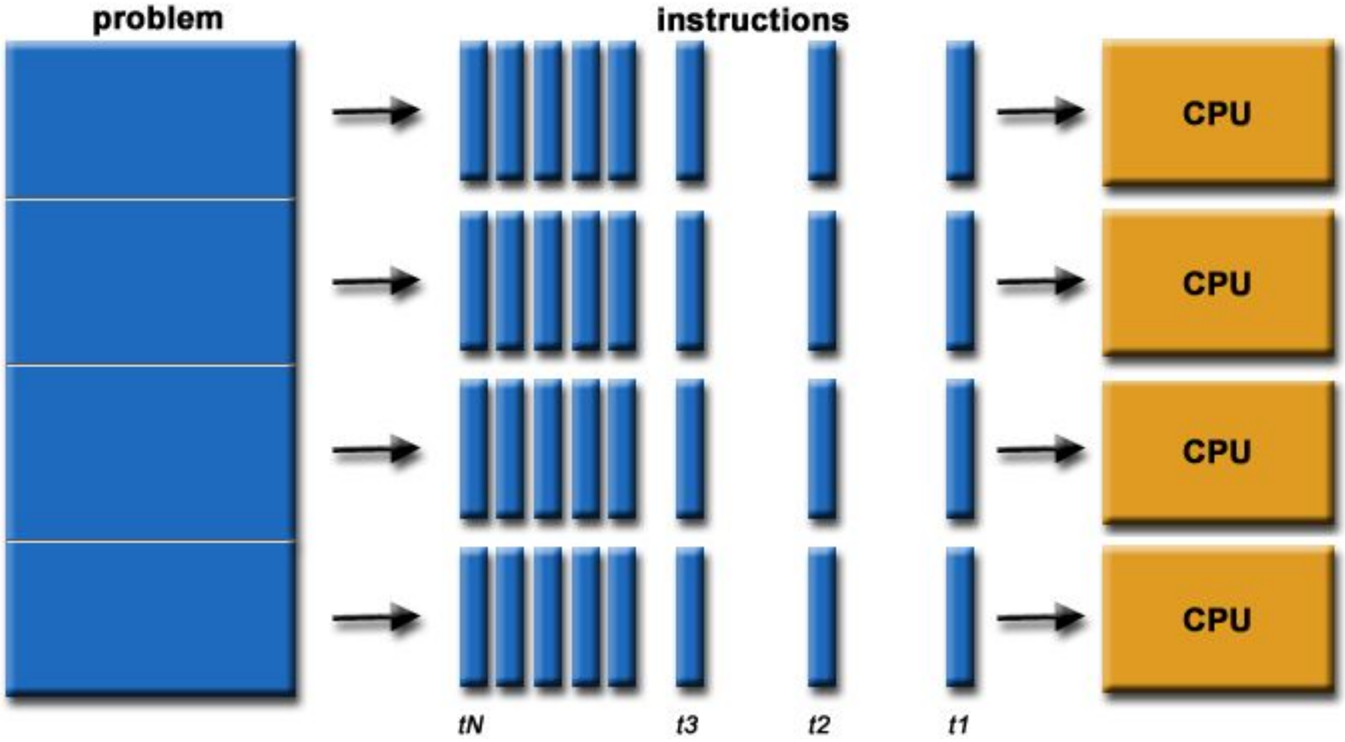

Parallelized program

Why should we learn how to use parallelism?

Rationale for parallelism

- The obvious goal for parallelism is to speed up the computation, via multiple processors
- Traditionally, parallel computation has been "high end," reserved for the realm of supercomputers
- Now, parallelism is mainstream…
    - Clusters themselves are mainstream
    - Multicore processors in your pocket

Rationale for parallelism

- Parallelism…
  - Saves time and money by solving problems faster
  - Allows us to solve larger instances of problems in the same amount of time
  - Allows use of non-local resources, e.g., SETI@Home
- Moore's law may be reaching its limit, so parallelism may be the way to continue to achieve improved performance

What are some different parallel computing architectures?

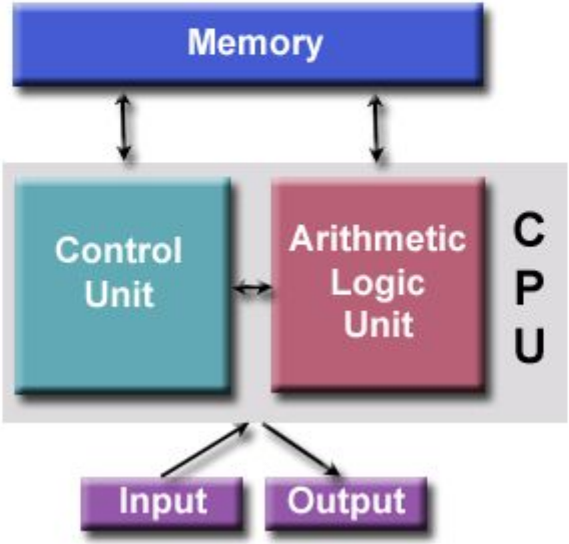

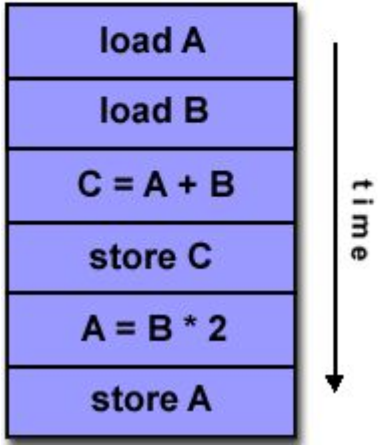

Classic von Neumann architecture

Parallel computing architectures

- Parallel architectures still retain the basic von Neumann scheme, but with multiple copies of some of the parts (CPU, memory, etc.)
- Parallel architectures can (still!) be classified with Flynn's Taxonomy, circa 1966
  - SISD -- single instruction, single data (one CPU)
  - SIMD -- single instruction, multiple data
  - MISD -- multiple instruction, single data
  - MIMD -- multiple instruction, multiple data

Parallel computing architectures

SIMD: Single Instruction, Multiple Data

All processors execute same instruction on a given clock cycle, but each may operate on a different data element; good for things like graphics / image processing

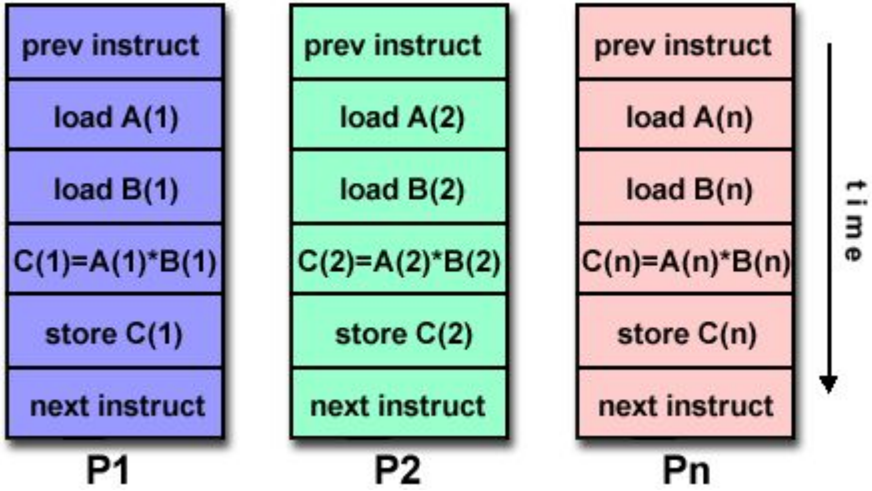

Parallel computing architectures

MISD: Multiple Instruction, Single Data

Each processor operates on same data independently, via separate instruction streams

Only a few research machines have applied this model

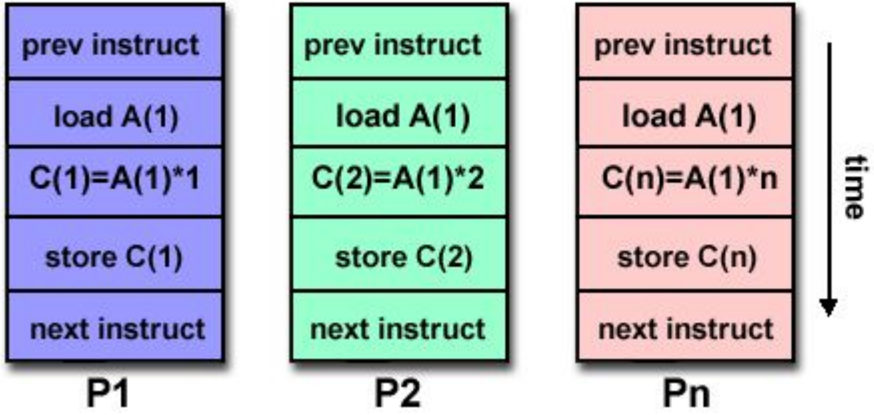

Parallel computing architectures

MIMD: Multiple Instruction, Multiple Data

Each processor may have different instruction stream

Each processor may be working on different data

Most common type today: Supercomputers, clusters, multicore PCs

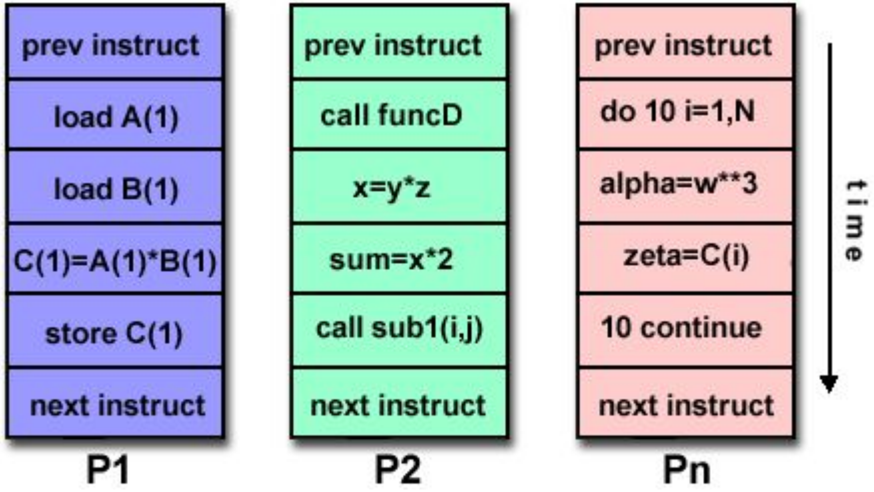

Parallel computing architectures

- What about memory? Can be either:
  - Shared: All CPUs have direct access to physical memory
  - Distributed: Network-based memory access
    - Node can only see its local memory
    - Must go over the network to access other nodes' memory

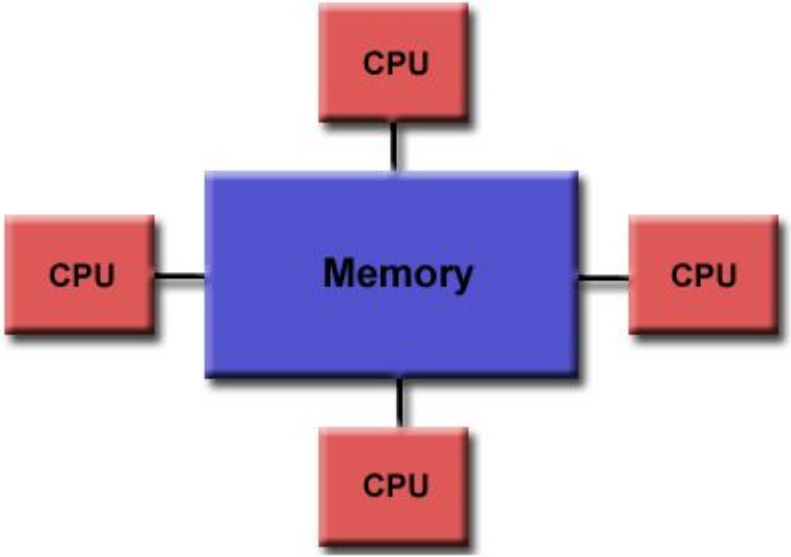

Uniform Memory Access (e.g., multicore CPUs)

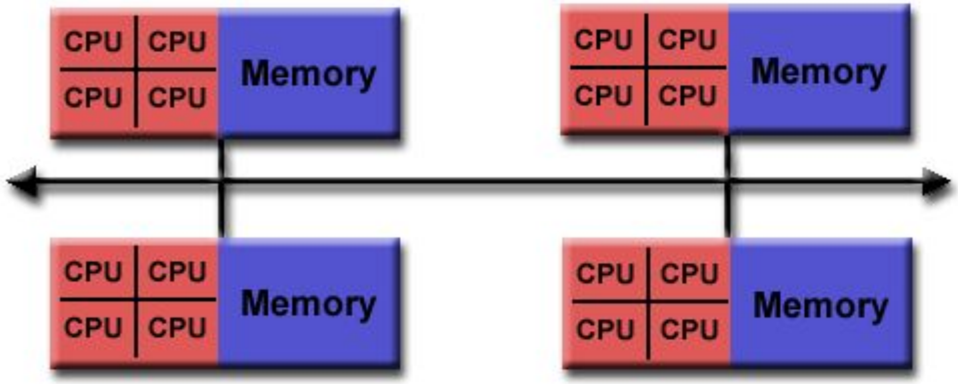

Hybrid distributed / shared (e.g., supercomputers / clusters)

How do we design parallelized programs?

Design of parallel programs

- High Throughput Computing (HTC)
  - Program is not parallelized
  - Same program runs on many nodes, each node acting on a separate piece of data
  - Nodes do not have to communicate with each other
  - E.g., OpenCV code operating on hundreds or thousands of images at the same time
- High Performance Computing (HPC)
  - Program is parallelized
  - One program uses multiple nodes at the same time
  - Communication between nodes is required
  - E.g., each node operating on a separate portion of a single, really huge image

Design of parallel programs (HPC)

- There are some tools that attempt to automatically parallelize your code, but the technology is still nascent / immature
- Manual parallelization is still the order of the day

Design of parallel programs (HPC)

- Understand your problem and any existing algorithms to solve it
    - Can the problem even be parallelized?
    - Where are the “hotspots” in existing algorithms (maybe use a profiler to find out)
    - What are potential bottlenecks, like I/O?
    - What data dependencies are there?

Design of parallel programs (HPC)

- Partitioning
  - Break problem into chunks that can be done in parallel, either by data or function
    - Data example: Image filtering
    - Function example: Multi-stage, pipelined signal filter

Design of parallel programs (HPC)

- Understand communications requirements
  - “Embarrassingly parallel” programs need little
  - But if communications are required between nodes, consider carefully
    - Communication over the network is SLOW, relatively speaking -- it’s the bottleneck

Design of parallel programs (HPC)

- Understand what synchronization you'll need
  - How do different processes manage access to shared resources?
- Try to balance the load
  - Keep all the CPUs equally busy all the time, ideally
- Understand limits / costs
  - What is actual speedup? Is it worth it?

Monte Carlo estimation of π
