# Supplementary material for: Digital imaging and vision analysis in science project improves the self-efficacy and skill of undergraduate students in computational work
Source: PLoS One. 2021 May 5;16(5):e0241946. doi: 10.1371/journal.pone.0241946 (PMC8099079; doi:10.1371/journal.pone.0241946)
Supplement: S7 File — (PDF) [file pone.0241946.s007.pdf]

BIO371 DIVAS II Burning Ship Fractal Project

Fall 2019

March 28, 2019

1 Introduction

In this assignment you will create a uni-processor and then a parallelized Python program to create an image of the *Burning Ship* fractal.

A *fractal*, as defined by Benoit Mandelbrot, is a "a rough or fragmented geometric shape that can be split into parts, each of which is (at least approximately) a reduced-size copy of the whole." Mandelbrot coined the term "fractal" in the 1970s, and one of the most famous fractals is the Mandelbrot set, as shown in the following images.

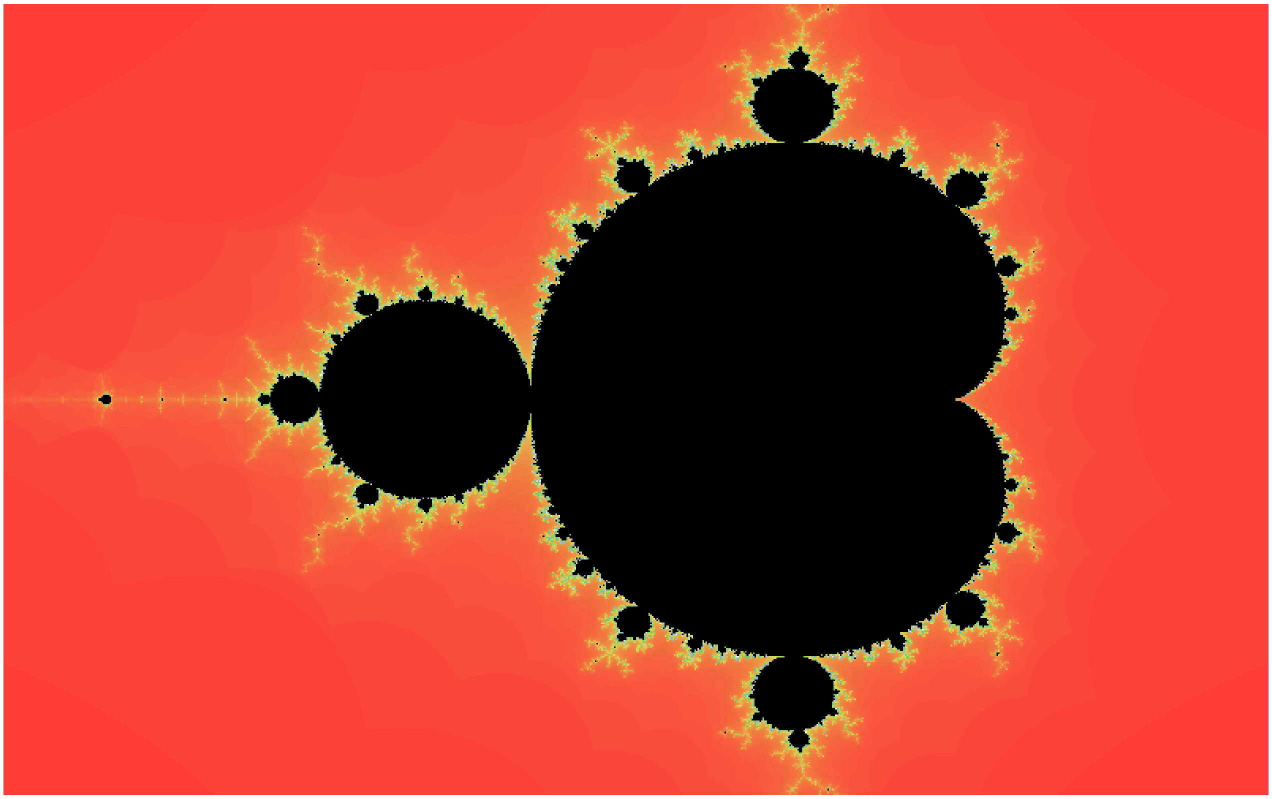

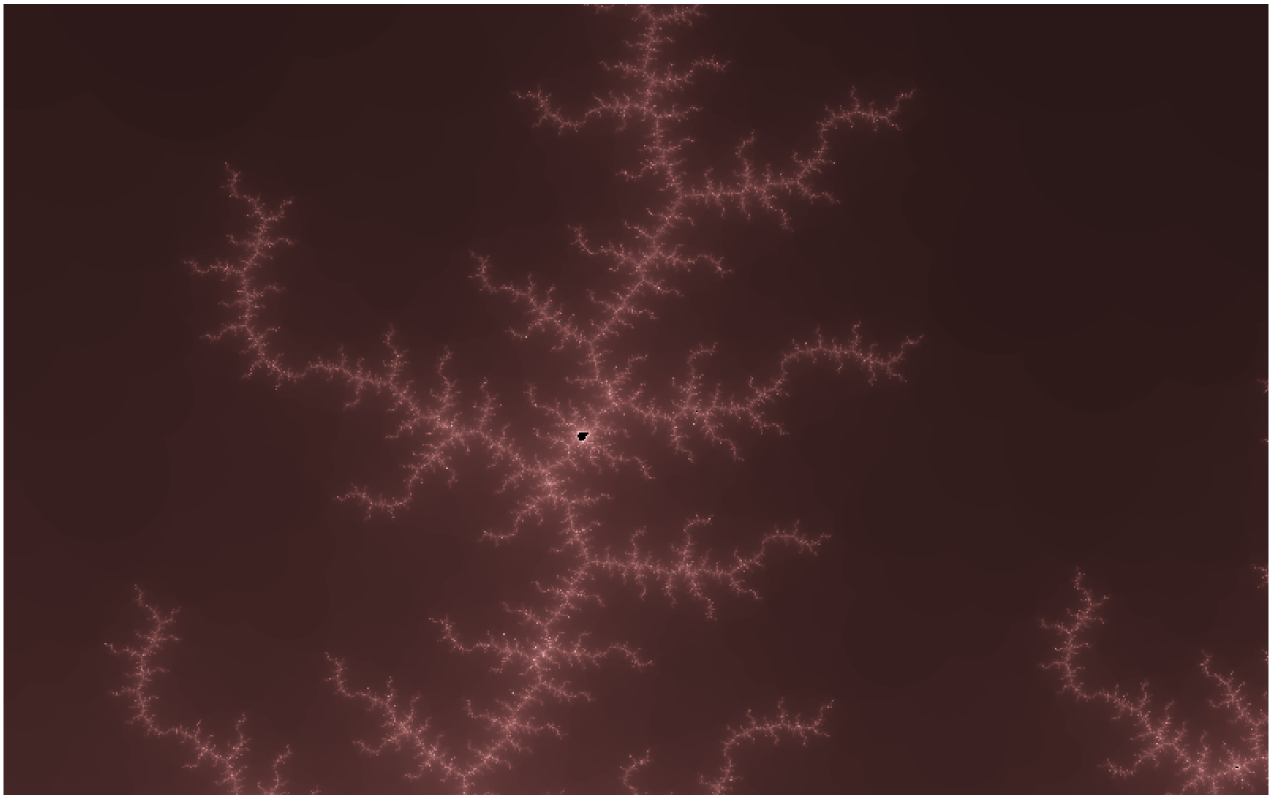

You will create images of a different fractal, namely, the Burning Ship fractal. Here are several images showing the fractal.

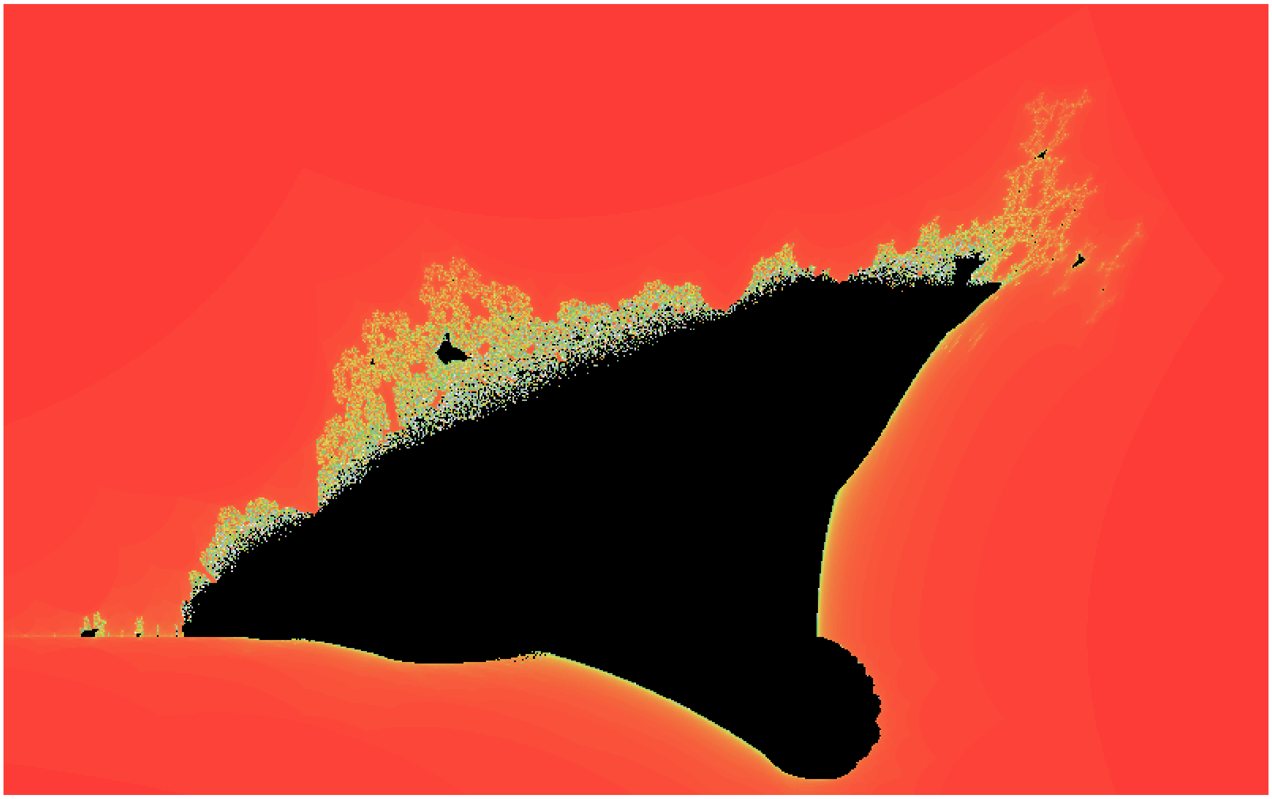

$(x_0, y_0) = (-2, -2)\,; (x_1, y_y) = (1.5, 0.5)$

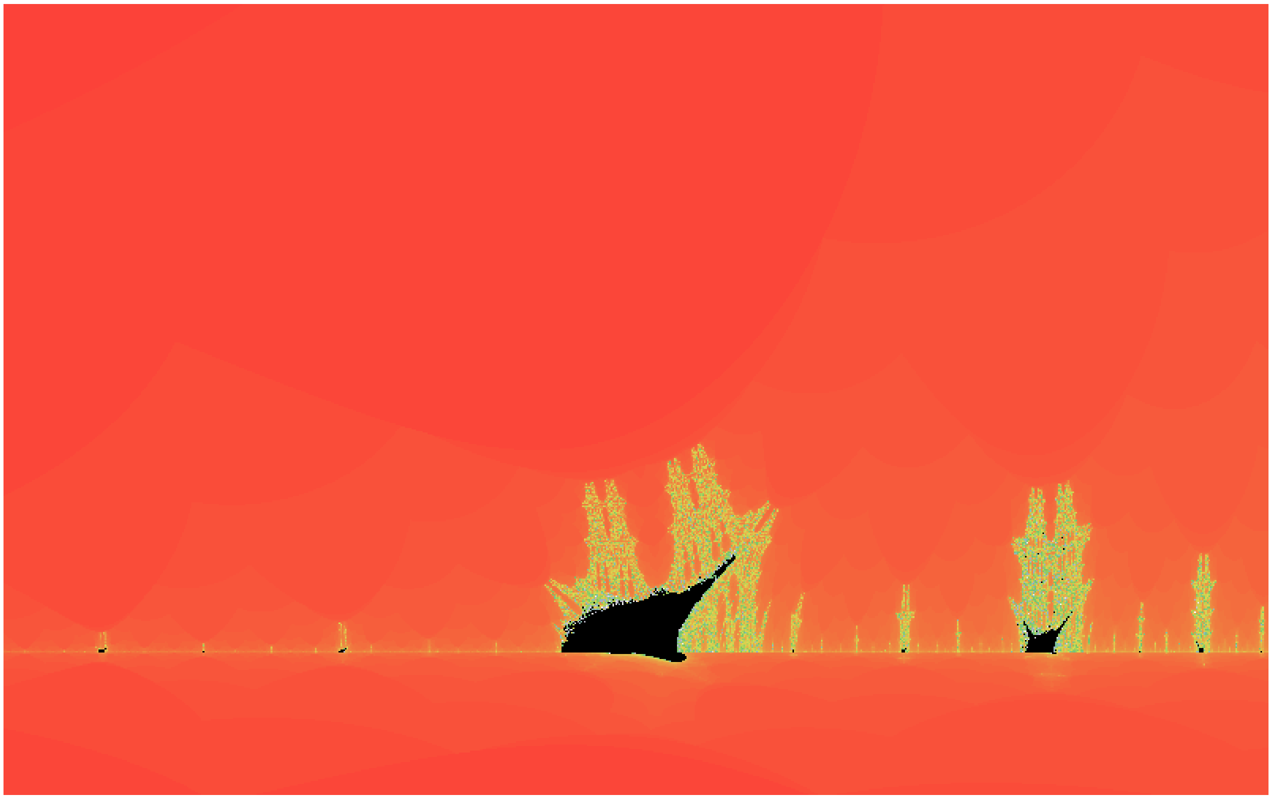

$(x_0, y_0) = (-1.974, -0.2465)\,;(x_1, y_y) = (-1.553, 0.05411)$

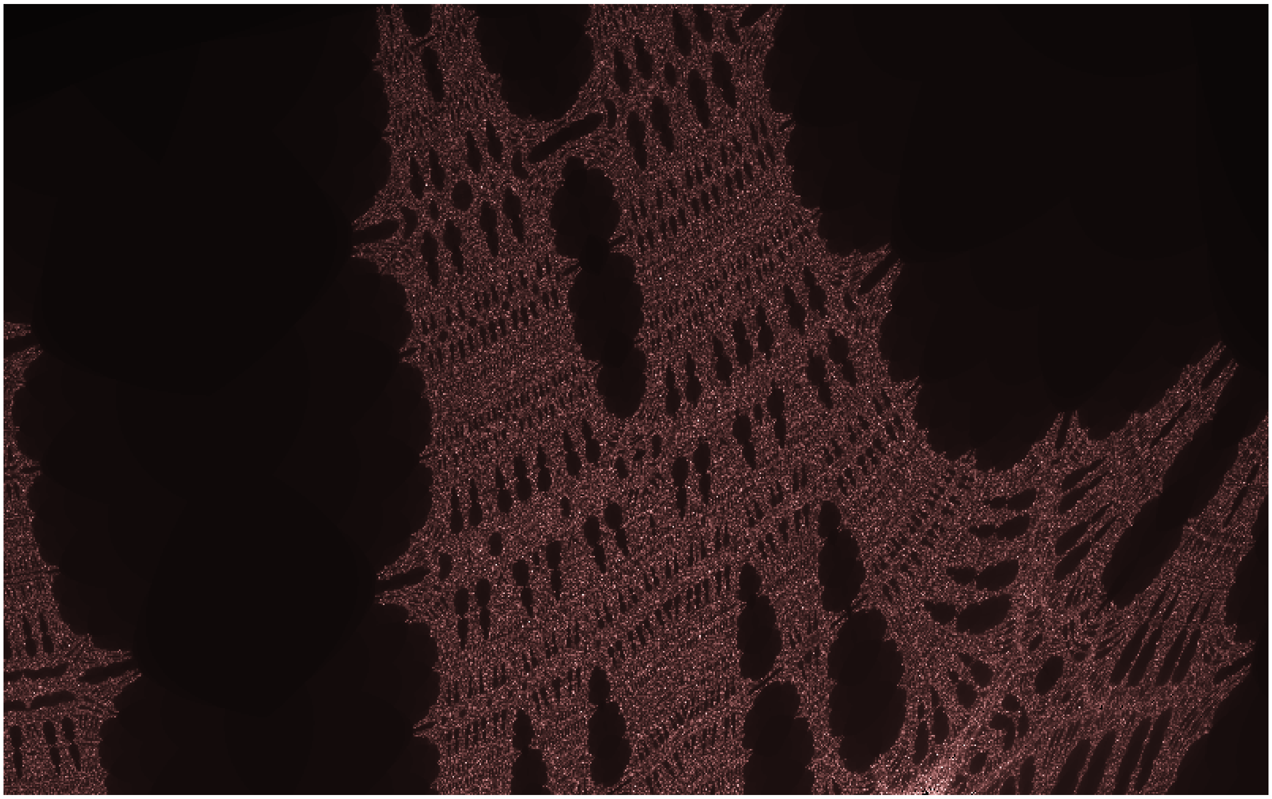

$(x_0, y_0) = (-1.768, -0.073)\,;(x_1, y_y) = (-1.717, -0.03686)$

The overall idea involved in drawing the fractal is quite simple: for each pixel in the image, your program will calculate the value of some mathematical function. Based on the results of that calculation,

you set the color of the pixel. Of course, as they say, the devil is in the details.

2 Uniprocessor version

Your first program will run on your own computer, without any parallelization. Design the program to read the image width, image height, x_0, y_0, x_1, x_2, and output filename from the command line. For example, to an image similar to the first Burning Ship shown above, we would execute the following command:

```
python burning-ship.py 800 600 -2 -2 1.5 0.5 bs1.png
```

One issue you will confront is that the coordinates of each pixel need to be translated to an (x, y) point before making the calculations. Consider this diagram:

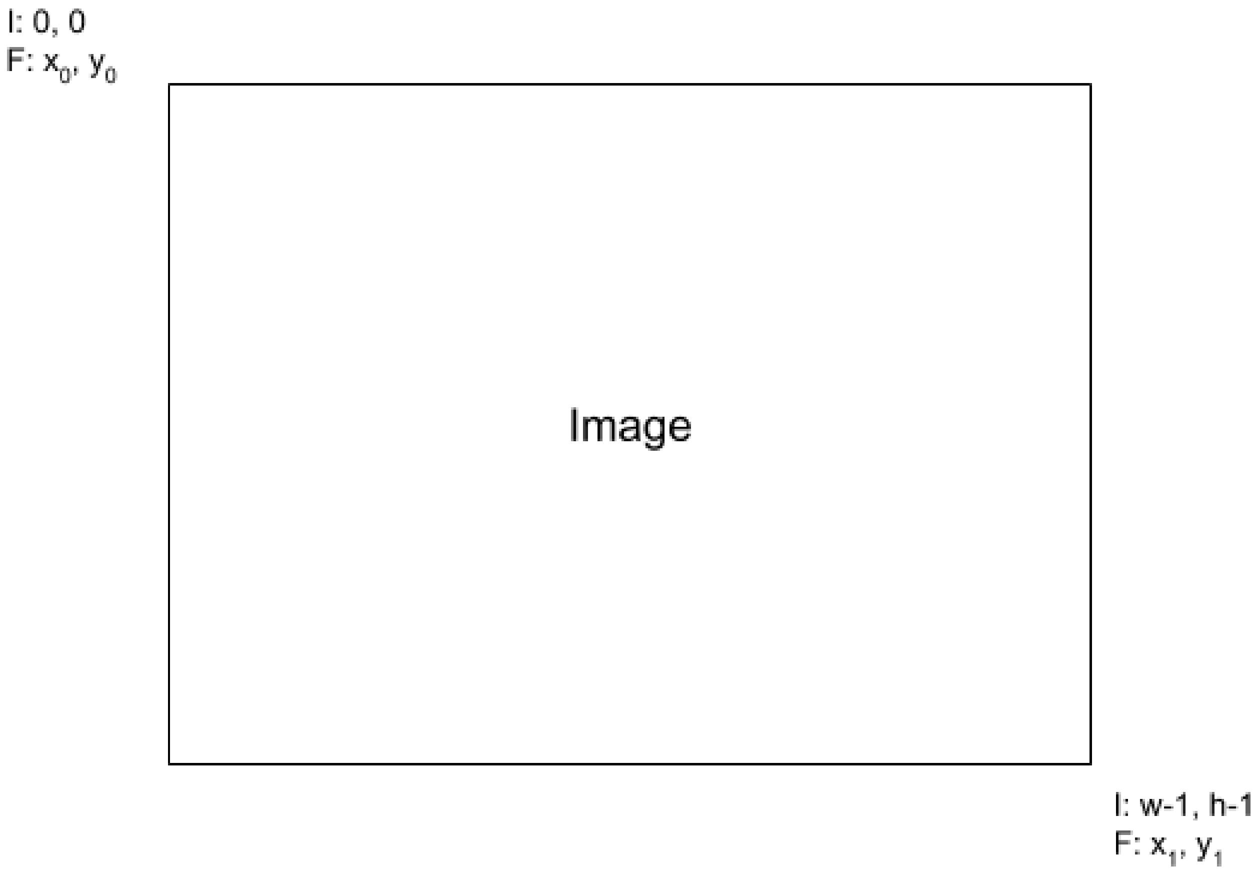

The rectangle represents your image, with coordinates shown for the upper left and lower right pixel. The top line of each coordinate label shows image coordinates, while the lower line of each shows corresponding real numbers for the fractal. For each pixel in the image, we need a way to convert the pixel coordinates to real numbers in the specified real range. For the first Burning Ship fractal image above, the upper left corner is at $(x_0, y_0) = (-2, -2)$, while the lower right corner coordinate is $(x_1, y_1) = (1.5, 0.5)$.

Here is a Python function you could use to do this translation:

```
'''
Map a value from the range [valLo, valHi] to [outLo, outHi].
```

```
'
' param val Value to map
' param valLo Lowest value val could have
' param valHi Highest value val could have
' param outLo Lowest value output could have
' param outHi Higest value output could have
'''
def mapToRange(val, valLo, valHi, outLo, outHi):
    return outLo + ((outHi - outLo) / (valHi - valLo)) * (val - valLo)
```

If the pixel you are currently processing is at row, col, then the function call

$$x = \text{mapToRange}(\text{row}, 0, w - 1, -2, 1.5)$$

would map the image row value to the correct fractal value x.

So, your program will have nested **for** loops to go through each pixel in the image. Inside the innermost loop you will have two calls to mapToRange() to get the (x, y) fractal coordinate.

Once you have an (x, y) point, you should calculate the value of the fractal at that point. Create a function named testPoint(x, y). Inside the function, create three float variables, a, ta, and a, each initialized to 0.0. Also create an integer variable i, initialized to 0. Then, execute the following statements in a **while** loop:

```
a = abs(a)
b = abs(b)
ta = a**2 - b**2 + x
b = 2 * a * b + y
a = ta
i += 1
```

If the absolute value of a or b ever gets to be larger than 2, end the loop and return the value of i. On the other hand, if the absolute values of a and b stay small, end the loop when the value of i reaches 255, and return i.

After calling the testPoint(x, y) function, you have an integer value between 0 and 255 corresponding to the real point (x, y), which corresponds to a pixel in the display window. You should choose a color for the pixel based on the integer value. For example, a simple grayscale color scheme would make the pixel black if the return value is 255, or a color with each component set to the return value otherwise. Experiment with different coloring schemes until you get one that you like.

The image you will ultimately save is an OpenCV image (i.e., 3D NumPy array).

3 Parallel version

The second part of the project is parallelize your code, using MPI, to run on Onyx. More details will be added to this section in the near future.
