# Supplementary material for: Digital imaging and vision analysis in science project improves the self-efficacy and skill of undergraduate students in computational work
Source: PLoS One. 2021 May 5;16(5):e0241946. doi: 10.1371/journal.pone.0241946 (PMC8099079; doi:10.1371/journal.pone.0241946)
Supplement: S8 File — (PDF) [file pone.0241946.s008.pdf]

Project description
The data set below is a live/dead assay for c elegans. Dead worms are straight rods while live worms are wavy. There is ground-truth data provided with the images that can be used to validate measurements. There is good variety in these images. This data set will challenge scholars to use the tools they've already learned about in news ways and potentially adopt new ones.

https://data.broadinstitute.org/bbbc/BBBC010/

Schedule
Each week: Meet each day at 9AM except for Thursday. Mondays will be used for overview and problem framing. Tuesday and Wednesday will be short 'stand up' meetings to set goals for that day's code. Code should be pushed by the end of the day Wednesday for review. Thursday at 1PM will be a code review day. Each pair should bring a diagram of the code from each of the other groups to the review. On Friday, code for that week can be cleaned up and edited based on the review discussion. This day is also used to think about the next problem within the project the pair would like to solve, which should be brought to Monday's meeting.

Side Projects
Depending on the time needed for pair programming projects, scholars can work on 'side projects'. Some of the side projects students worked on included:

- Programming Raspberry Pis to image and analyze time-lapsed images of brome grass for a teacher workshop
- Developing an image-based method for measuring chemotaxis
- Developing colorimetric sensors for detection of sugars in root exudates
- Developing a semi-automated approach for measuring growth of maize seedlings

The rationale for adding side projects (as scholars had time for them) is to provide a break from the pair programming project, which could sometimes become fairly challenging. The scope of the work within each 'side project' was intentionally very well defined so that scholars found making progress achievable and manageable.
