# Supplementary material for: Digital imaging and vision analysis in science project improves the self-efficacy and skill of undergraduate students in computational work
Source: PLoS One. 2021 May 5;16(5):e0241946. doi: 10.1371/journal.pone.0241946 (PMC8099079; doi:10.1371/journal.pone.0241946)
Supplement: S9 File — (PDF) [file pone.0241946.s009.pdf]

DIVAS Career Path and Self Efficacy

Start of Block: Demographics and Intended Career

Q15 Enter your institutional email address

____

Q32 Enter the current term

▼ Spring 2020 ... Summer 2021

Q36 Indicate your current position

High School Student

Undergraduate Student

Graduate Student

Postoctoral Scholar

Faculty/Staff

Other

Skip To: End of Block If Q36 = Faculty/Staff

Skip To: End of Block If Q36 = Other

Skip To: End of Block If Q36 = Graduate Student

Skip To: End of Block If Q36 = Postoctoral Scholar

Display This Question:

If Q36 = Undergraduate Student

Q2 When do you expect to or when did you graduate from college?
Year

Month

▼ 2020 ... Other ~ Other

Display This Question:

If Q36 = Undergraduate Student

Q1 How many semesters of college after completing high school have you completed? In other words, do not include college credit gained as a high school student.

▼ less than 1 ... more than 8

Display This Question:

If Q36 = Undergraduate Student

Q33 Are you a DIVA scholar?

Yes

No

Q34 What class or event are you in that you are taking this survey for?

Coding Workshop

CHM 125 or 126

BIO 356

DIVAS Seminar I

DIVAS Seminar II

Summer Research Project

UNL Software Carpentry Workshop

Other

Display This Question:

If Q34 = Other

Q35 Indicate the class or event that is associated with this survey

____

Page Break

Q3 What is your intended or actual major(s)? Use the 'ctrl' key to select multiple.

- Biology
- Biochemistry
- Chemistry
- Computer Science
- Engineering
- Environmental Science
- Health and Society
- Math
- Physical Education
- Physics
- Psychology
- Other

Display This Question:

If Q3 = Other

Q4 Indicate other major(s)

____

Q5 What is your intended or minor(s) (if any)? Use the 'ctrl' key to select multiple.

- Biology
- Chemistry
- Comutational Science
- Computational Thinking
- Math
- Physics
- Psychology
- Sociology
- Spanish

Other
None

Display This Question:

If Q5 = Other

Q6 Indicate other minor(s)

____

Q7 What is your intended (or actual) career? Please be specific.

____Q8 In whatever career I choose, I would like to work in a job that uses computer science applications, programming, or computational thinking.

Strongly agree
Agree
Neutral
Disagree
Strongly disagree

Display This Question:

If Q36 = High School Student

Or Q36 = Undergraduate Student

Or Q36 = Graduate Student

Or Q36 = Postoctoral Scholar

Q9 I would like to spend my life working with computer science applications, programming, or computational thinking.

Strongly agree
Agree
Neutral
Disagree
Strongly disagree

Display This Question:

If Q36 = High School Student

Or Q36 = Undergraduate Student

Or Q36 = Graduate Student

Or Q36 = Postoctoral Scholar

Q10 In my career, I would like to work with projects that involve a lot of computer science applications, programming, or computational thinking.

- Strongly agree
- Agree
- Neutral
- Disagree
- Strongly disagree

Display This Question:

If Q36 = High School Student

Or Q36 = Undergraduate Student

Or Q36 = Graduate Student

Or Q36 = Postoctoral Scholar

Q11 How much do you know about careers and jobs that involve computer science applications, programming, or computational thinking?

- A lot
- Some
- Not a lot
- Very little

Display This Question:

If Q36 = High School Student

Or Q36 = Undergraduate Student

Or Q36 = Graduate Student

Or Q36 = Postoctoral Scholar

Q12 How much do you know about what has to be done in order to get a job in my chosen career that uses computer science applications, programming, or computational thinking?

A lot
Some
Not a lot
Very little

Display This Question:

If Q36 = High School Student

Or Q36 = Undergraduate Student

Or Q36 = Graduate Student

Or Q36 = Postoctoral Scholar

Q13 How much do you know about where to find information about jobs and careers that use computer science applications, programming, or computational thinking?

A lot
Some
Not a lot
Very little

Display This Question:

If Q36 = High School Student

Or Q36 = Undergraduate Student

Or Q36 = Graduate Student

Or Q36 = Postoctoral Scholar

Q14 How much do you know about how much computer science applications, programming, or computational thinking are used in career(s) you have chosen or are considering?

A lot

Some
Not a lot
Very little

Page Break

End of Block: Career Outlook

Start of Block: Self-efficacy

Q17 Please rate how confident you are about your knowledge of or ability to do each of the following on a scale from 0 (Completely Unconfident) to 100 (Completely Confident). The scale descriptors (e.g., Very Unconfident, Somewhat Confident, etc.) are provided as general descriptors only. Except for "Completely Unconfident" and "Completely Confident" which represent 0 and 100 respectively, the descriptors are not tied to a specific number on the numeric scale. You may put down any number between 0 and 100. Rate only your current level of knowledge or ability; what you know or are able to do right now at this point in time. Do not consider how much you might know or be able to do at some future time.

Q18 Your knowledge of computational thinking.

____

Q21 Your ability to use computational algorithms to solve problems in your field.

____

Q22 Your ability to use objects in writing computer programs.

____

Q23 Your ability to generate new computational solutions to problems in your field.

Q24 Your ability to use computer and software tools in your field.

Q25 Your ability to write recursive routines in computer programs.

Q26 Your ability to think of novel ways of doing things in your field.

Q27 Your ability to conceptualize data in your field in ways that can be analyzed computationally.

Q28 Your ability to decompose problems in ways that can be solved algorithmically.

Q29 Your ability to systematically approach a problem to find a solution.

Q30 Your ability to view problems within your field in different ways.

Q31 Your ability to think of multiple ways to approach solving a problem computationally.

End of Block: Self-efficacy
