# Supplementary material for: Digital imaging and vision analysis in science project improves the self-efficacy and skill of undergraduate students in computational work
Source: PLoS One. 2021 May 5;16(5):e0241946. doi: 10.1371/journal.pone.0241946 (PMC8099079; doi:10.1371/journal.pone.0241946)
Supplement: S10 File — This rubric was used in year 1 of the study. (PDF) [file pone.0241946.s010.pdf]

Computational Thinking Rubric

RADIS phase	CT Skill	Proficient (3)	Progressing (2)	Novice (1)
Recognize	*1. What is the problem we need to solve?*	• Recognizes the problem<br>• Can write a clear, concise, and complete problem statement	• Recognizes the problem<br>• Problem statement needs improvement in clarity, conciseness, and / or completeness	• Does not recognize the problem<br>• Unable to write a clear, concise, and complete problem statement
	2. Collect data	• Knows what data is required to solve the problem<br>• Knows where the data is and how to access it	• Knows what data is required to solve the problem<br>• Does not know where the data is and / or how to access it	• Does not know what data is required to solve the problem<br>• Does not know where the data is or how to find it

RADIS phase	CT Skill	Proficient	Progressing	Novice
Analyze	*3. Are multiple solutions possible (including the current process)? Which is better?*	• Recognizes that multiple solutions are plausible<br>• Selects a solution based on criteria such as efficiency, ease of use, cost, etc.	• Recognizes that multiple solutions exist<br>• Selects an implausible or very suboptimal solution<br>• Cannot fully explain rationale for choosing a solution	• Sees no possible solution<br>• Cannot explain rationale for choosing a solution
	4. What available computerized tools could be used? Which is better?	• Recognizes that multiple tools are plausible<br>• Selects a tool based on criteria such as efficiency, ease of use, cost, etc.	• Recognizes that multiple tools exist<br>• Selects an implausible or very suboptimal tool<br>• Cannot fully explain rationale for choosing a tool	• Sees no possible tool to solve the problem<br>• Cannot explain rationale for choosing a tool
	5. Analyze data	• Recognizes characteristics of the data that lead to a “natural” selection of a solution and tool	• Recognizes that characteristics of the data may drive the selection of a solution and tool<br>• Focuses on the wrong characteristics and / or makes a flawed selection of a solution and / or tool based on the characteristics	• Unable to recognize characteristics of the data that drive selection of a tool and solution

RADIS phase	CT Skill	Proficient	Progressing	Novice
Analyze	6. Abstraction / modeling / representation	• Understands that abstract representation of data is required<br>• Able to understand, select, and communicate how data will be represented for the given solution and tool	• Understands that abstract representation of data is required<br>• Misidentifies appropriate representation for the given solution and tool<br>• Cannot effectively communicate representation	• Does not understand that abstract representation of data is required<br>• Unable to understand, select, or communicate how data will be represented for the given solution and tool
	7. Decomposition	• Able to break the large problem down into smaller subproblems<br>• Subproblem solutions can be combined to solve the larger problem	• Able to break the large problem down into smaller subproblems, but the subproblems may be inappropriate / unnecessary<br>• Solving subproblems does not lead to overall solution	• Unable to break problem down into smaller subproblems<br>• Solving subproblems does not lead to overall solution

RADIS phase	CT Skill	Proficient	Progressing	Novice
Design	8. Algorithmic thinking (AT)	• Can create a step-by-step process that solves the problem, using the selected solution and tool	• Creates a step-by-step process intended to solve the problem using the selected solution and tool<br>• Process contains unrecognized errors that will prevent a solution once implemented	• Cannot create a step-by-step process that solves the problem using the selected solution and tool
	9. AT / sequence	• Selects and orders a sequence of steps or commands that produces the targeted outcome	• Selects an ordered sequence of steps or commands, but due to minor errors the targeted outcome is not achieved	• Cannot select an ordered sequence of steps or commands to produce the targeted outcome
	10. AT / selection	• Recognizes the need for selection; and can produce a series of steps or commands lead to the targeted outcome	• Recognizes the need for selection; but due to minor errors the steps or commands do not lead to the targeted outcome	• Does not recognize the need for selection -- doing one thing or another, based on input -- to solve the problem
	11. AT / iteration	• Recognizes the need for iteration; and can produce a series of steps or commands lead to the targeted outcome	• Recognizes the need for iteration; but due to minor errors the steps or commands do not lead to the targeted outcome	• Does not recognize the need for iteration -- doing the same step or series of steps multiple times -- to solve the problem

RADIS phase	CT Skill	Proficient	Progressing	Novice
Implementation	12. Organizes data	• Organizes data in a manner such that access via computerized tools is easy	• Some effort at organization, but the chosen scheme does not favor the tool chosen for the solution	• Cannot organize data to make access via computerized tools easy
	13. Creates solution	• Implements the solution using the specified tool(s)<br>• Implementation correctly solves the problem	• Implements the solution using the specified tool(s)<br>• Minor implementation issues prevent correct solution	• Cannot implement the solution using the specified tool(s)<br>• Major implementation issues prevent correct solution
	14. Testing/Debugging	• Refines algorithm multiple times using testing / debugging processes.	• Evidence of preliminary testing / refining of algorithm. Some data irregularities not accounted for.	• No evidence of efforts to refine algorithm or account for pattern irregularities
	15. Reusing and Remixing	• Clear evidence of using previous or searched knowledge to simplify processes / algorithm<br>• Understands why the reused material is appropriate for this solution	• Some evidence of using previous or searched knowledge to simplify processes / algorithm<br>• Some understanding of why the reused material is appropriate for this solution	• No evidence of using prior or searched knowledge in task<br>• Evidence of reuse or remixing, but it is not appropriate for this solution
