# Supplementary material for: Digital imaging and vision analysis in science project improves the self-efficacy and skill of undergraduate students in computational work
Source: PLoS One. 2021 May 5;16(5):e0241946. doi: 10.1371/journal.pone.0241946 (PMC8099079; doi:10.1371/journal.pone.0241946)
Supplement: S12 File — This rubric was used in year 3 of the study. (PDF) [file pone.0241946.s012.pdf]

DIVAS Computational Thinking Rubric v. 4

RADIS Phase	CT Skill	5	4	3	2	1
**Recognize**	**1.A. What is the problem we need to solve? (recognition)**	The solution demonstrates that the problem is recognized, including most of the deeper subtleties	The problem is recognized, including some deeper subtleties	The problem is recognized at a surface level	Some of the surface-level parts of the problem are recognized	The problem is not recognized, even at a surface level
	**1.B. What is the problem we need to solve? (expression)**	Can write a clear, concise, and complete problem statement that accurately describes the problem	Can write a clear and concise problem statement that accurately describes the problem, but needs some improvement in clarity and / or conciseness	Can write a mostly clear and concise problem statement that describes the significant aspects of the problem, but needs improvement in clarity, conciseness, and / or completeness	Can write a problem statement, but omits significant elements of the problem, and / or needs significant improvement in clarity	Unable to write a problem statement that accurately captures the problem
	**2.A. Data collection (understanding)**	Recognizes what data is required to solve the problem, and understands the implications involved in storing / accessing / transferring the data	Recognizes what data is required to solve the problem, and understands some of the implications involved in storing / accessing / transferring the data	Recognizes what data is required to solve the problem	Incompletely recognizes what data is required to solve the problem	Does not know what data is required to solve the problem
	**2.B. Data collection (locating / sourcing)**	Knows where the data is stored and how to access the data in an efficient manner	Knows where the data is stored, but may access the data in an inefficient manner	Knows where the data is stored, but accesses it in a significantly inefficient manner	Knows where the data is stored, but is unable to effectively access it	Does not know where the data is stored or how it would be accessed

Recognize section total score: _____

RADIS Phase	CT Skill	5	4	3	2	1
**Analyze**	**3.A. Evaluation of solutions (identification)**	Can identify multiple plausible solutions to the problem	Can identify multiple solutions to the problem, but some of the solutions are implausible	Can identify a plausible solution to the problem	Identifies a potential solution, but does not recognize its implausibility	Cannot identify any possible solutions to the problem
	**3.B. Evaluation of solutions (data analysis)**	Recognizes characteristics of the data that lead to a selection of an efficient solution	Recognizes most characteristics of the data that would lead to an efficient solution. Solution is not optimally efficient.	Recognizes some characteristics of the data that lead to a solution, but the solution is significantly less efficient than other options	Misidentifies the important characteristics of the data, leading to an implausible or very inefficient solution	Unable to recognize characteristics of the data that lead to a selection of a solution
	**3.C. Evaluation of solutions (selection)**<br><br>***Requires a record of different solutions considered***	Selects a workable solution to the problem based on criteria such as efficiency, cost, ease of use, etc.	Selects a workable solution to the problem based on criteria such as efficiency, cost, ease of use, etc., but does not select an alternate solution that meets the criteria better	Selects a workable solution, with some consideration for criteria such as efficiency, cost, ease of use, etc.	Selects a workable solution, but seemingly at random, without thought about criteria such as efficiency, cost, ease of use, etc.	Does not select a workable solution to the problem
	**4. Data representation**	Represents the required data in the solution in an optimal manner	Represents the required data in the solution	Represents the required data in the solution, but in an inefficient manner	Partially represents the required data in the solution	Cannot appropriately represent the required data in the solution
	**5. Decomposition**	Decomposes the problem into appropriate, fully cohesive (self-contained, single-purpose) component subproblems	Decomposes the problem into appropriate, partially cohesive (self-contained, single-purpose) component subproblems	Decomposes the problem into appropriate component subproblems	Decomposes the problem into inappropriate or unnecessary component subproblems	Cannot decompose the problem into component subproblems

Analyze section total score: _____

RADIS Phase	CT Skill	5	4	3	2	1
**Design**	**6.A. Algorithmic thinking (sequence)**	Designs an optimal, step-by-step process that solves the problem	Designs suboptimal, step-by-step process that solves the problem	Designs an inefficient, step-by-step process that solves the problem, or a step-by-step process that nearly solves the problem	Designs a process using steps that partially solves the problem. Step order and/or operations within steps contain/s errors.	Cannot design a step-by-step process that solves the problem
	**6.B. Algorithmic thinking (selection)**	Utilizes selection in the design to improve the efficiency or simplicity of an otherwise correct algorithm	Recognizes the need for selection to solve the problem and correctly incorporates it into the design	Recognizes the need for selection to solve the problem and incorporates it into the design in a mostly correct manner	Recognizes the need for selection to solve the problem, but cannot incorporate it into the design	Does not recognize the need for selection to solve the problem
	**6.C. Algorithmic thinking (iteration)**	Utilizes iteration in the design to improve the efficiency or simplicity of an otherwise correct algorithm	Recognizes the need for iteration to solve the problem and correctly incorporates it into the design	Recognizes the need for iteration to solve the problem and incorporates it into the design in a mostly correct manner	Recognizes the need for iteration to solve the problem, but cannot incorporate it into the design	Does not recognize the need for iteration to solve the problem

Design section total score: _____

RADIS Phase	CT Skill	5	4	3	2	1
**Implement**	**7. Organizes data**	Organizes data so it can be optimally accessed by the implementation	Organizes data so it can be accessed by the implementation	Organizes data so it can be accessed by the implementation, but the organization causes the process to be inefficient	Makes some effort at organizing data so it can be accessed by the implementation, but organizational errors prevent a solution	Does not recognize the need to organize data to make it accessible by the implementation
	**8. Creates solution**	Implementation solves the problem in an optimal manner	Implementation solves the problem	Implementation solves the problem in an inefficient manner	Implementation of the solution does not solve the problem, due to logic errors	Implementation of the solution does not solve the problem, due to syntax errors
	**9. Refinement**	Clear evidence that the solution was refined to significantly improve simplicity and efficiency	Evidence that the simplicity and / or efficiency of the solution was improved via refinement	Evidence that the solution was refined, resulting in a slight improvement in efficiency or simplicity	Some evidence that the solution was refined, but the result is not an improvement in simplicity or efficiency	No evidence of efforts to refine the solution
	**10. Robustness**	The solution accounts for virtually all possible irregularities in input or data	The solution accounts for many possible irregularities in input or data	The solution accounts for the most likely irregularities in input or data	The solution accounts for some likely irregularities in input or data	The solution does not account for irregularities in input or data
	**11. Reuse**	Evidence of using prior or searched knowledge to improve the operation, efficiency, or simplicity of the solution, and evidence of understanding of why the reuse is appropriate	Evidence of using prior or searched knowledge to improve the operation, efficiency, or simplicity of the solution	Some evidence of using prior or searched knowledge to improve the solution, but more opportunities for reuse are evident	Evidence of using prior or searched knowledge in solution, but the reuse is not appropriate for the solution	No evidence of using prior or searched knowledge in solution

Implement section total score: _____
