# Supplementary material for: Digital imaging and vision analysis in science project improves the self-efficacy and skill of undergraduate students in computational work
Source: PLoS One. 2021 May 5;16(5):e0241946. doi: 10.1371/journal.pone.0241946 (PMC8099079; doi:10.1371/journal.pone.0241946)
Supplement: S13 File — (PDF) [file pone.0241946.s013.pdf]

This summarizes the differences between version 3 of the CT rubric and version 4, as of 6/18/2019.

The translation from the previous rubric to the new one is summed up in this table:

Item	Note
overall	Changed scale with ‘1’ as low and ‘5’ as high
1.A.	Changed level ‘5’ to ‘The solution demonstrates that the problem is recognized, including deeper subtleties’, changed level ‘4’ to ‘The problem is recognized, including some deeper subtleties’, changed level ‘3’ to ‘The problem is recognizedRecognizes the problem at a surface level’, changed level ‘2’ to ‘Some of the surface-level parts of the problem are recognized’, changed level ‘1’ to ‘The problem is not recognized, even at a surface level’
1.B.	
2.A.	
2.B.	
3.A.	
3.B.	Changed level 4 to ‘Recognizes most characteristics of the data that would lead to an efficient solution. Solution is not optimally efficient.’
3.C.	
4.	
5.	
6.A.	Added “, or a step-by-step process that nearly solves the problem” at the end of text in level 3, changed level 2 to ‘Designs a process using steps that partially solves the problem. Step order and/or operations within steps contain/s errors. ’
6.B.	
6.C.	
7.	
8.	
9.	

10.	
11.	
