# Supplementary material for: Digital imaging and vision analysis in science project improves the self-efficacy and skill of undergraduate students in computational work
Source: PLoS One. 2021 May 5;16(5):e0241946. doi: 10.1371/journal.pone.0241946 (PMC8099079; doi:10.1371/journal.pone.0241946)
Supplement: S14 File — (PDF) [file pone.0241946.s014.pdf]

This summarizes the differences between version 2 of the CT rubric and version 3, as of 1/24/2018.

V. 3 has five levels for each rubric row, versus three, and the value judgements have been removed from the levels. Rows have been separated, renumbered, etc., so that each row is hopefully only evaluating one skill. The translation from the previous rubric to the new one is summed up in this table:

V. 2 item	V. 3 item	Note
1.	1.A.	Split row 1 into two rows
	1.B.	
2.	2.A.	Split row 2 into two rows
	2.B.	
3.	3.A.	Split row 3 into three rows
	3.B.	
	3.C.	
4.	-	Removed; how are “solutions” and “tools” different, really?
5.	-	Folded into v. 3 item 3.B.
6.	4.	
7.	5.	
8.	6.A.	
9.	-	Removed; implied in 6.A.
10.	6.B.	
11.	6.C.	
12.	7.	
13.	8.	
14.	9.	Split 14 into “refinement” and “robustness,” which are different
	10.	

15.	11.	
