# Supplementary material for: Digital imaging and vision analysis in science project improves the self-efficacy and skill of undergraduate students in computational work
Source: PLoS One. 2021 May 5;16(5):e0241946. doi: 10.1371/journal.pone.0241946 (PMC8099079; doi:10.1371/journal.pone.0241946)
Supplement: S17 File — (PDF) [file pone.0241946.s017.pdf]

Cup castle prompt

Imagine there is a cup stacking robot that can build any particular arrangement of cups using specific commands that you give it. Shown below is a stack of cups at ‘0 Steps’ (Assume that there are an infinite number of cups at ‘0 Steps’), plus a few more cups at 2, 3, and 4 steps.

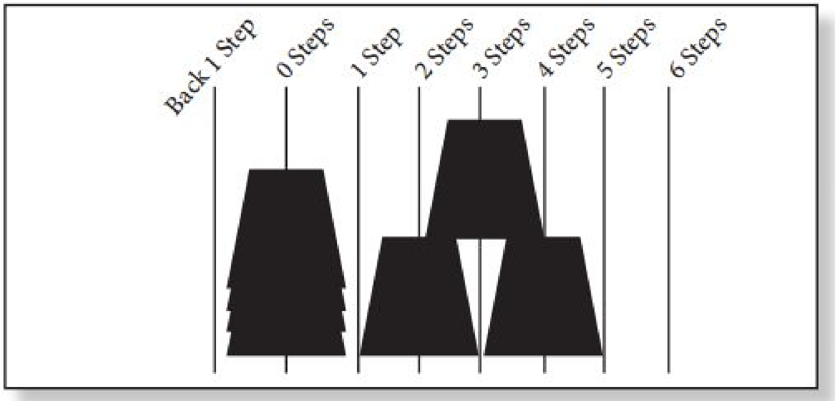

Step Guide

The commands the robot knows how to follow are:

↑ - Pick cup up

↓ - Put cup down

Picking a cup up raises it as high as it needs to go, and putting it down lowers it until it comes into contact with the table or another cup.

→ - Move ½ cup width to the right

← - Move ½ cup width to the left

↷ - Turn cup 90° clockwise (e.g. from 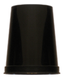 to 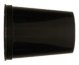)

↶ - Turn cup 90° counterclockwise (e.g. from 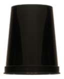 to 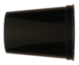)

For example, consider this cup castle:

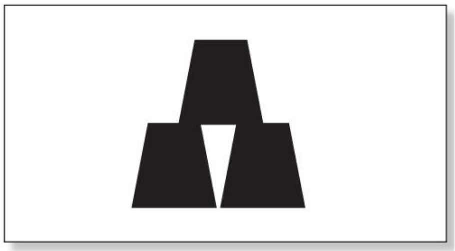

Here are one set of commands the robot could follow to create the three-cup configuration above.

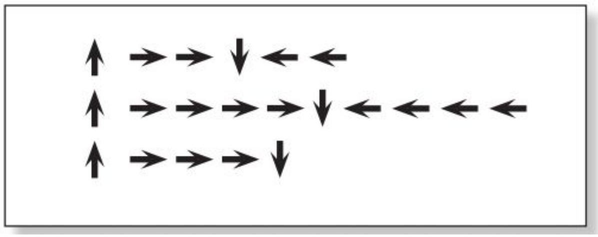

Now, it’s your turn to tell the robot what to do! Write a series of commands to create the following, 8-cup castle:

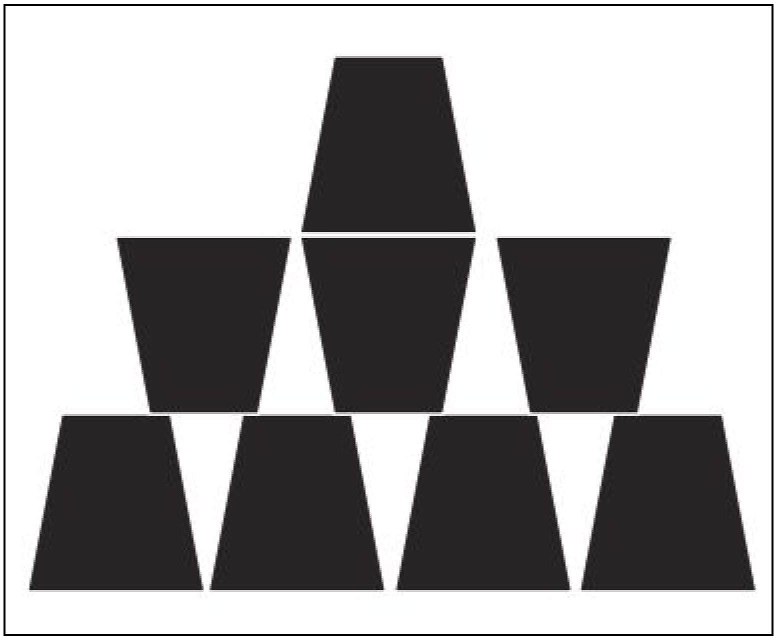

Write a set of commands that would create the configuration of cups below:

Looking at what you've written, try to reduce the number of commands without changing the final cup configuration in order to make the robot create the 8-cup castle with the fewest number of commands possible.

Write out your revised series that uses the fewest number of commands below:

Advanced only: You may define new commands and variables for the robot if you wish. Make sure any new commands or variables you define could be easily understood by someone with no prior experience, and also are likely to be executed by a robot.

Potential Follow up Questions:

1. Restate the problem your commands were designed to solve in your own words.

2. Which part of this task did you find most challenging, if any?

3. In words, describe the approach you took to solving this problem. How confident are you that your solution works?

4. How did you determine that your solution is correct? How does it compare to other possible solutions?

5. How efficient do you see your solution? Do you see places in your solution where it would be possible to reduce the number of commands?
