# Supplementary material for: Digital imaging and vision analysis in science project improves the self-efficacy and skill of undergraduate students in computational work
Source: PLoS One. 2021 May 5;16(5):e0241946. doi: 10.1371/journal.pone.0241946 (PMC8099079; doi:10.1371/journal.pone.0241946)
Supplement: Attachment — Submitted filename: PONE-D-20-33866_reviewer sw.docx [file pone.0241946.s023.docx]

PLOS ONE

Digital Imaging and Vision Analysis in Science Project improves the self-efficacy and skill of undergraduate students in computational work

--Manuscript Draft--

| Manuscript Number: | PONE-D-20-33866 |
| --- | --- |
| Article Type: | Research Article |
| Full Title: | Digital Imaging and Vision Analysis in Science Project improves the self-efficacy and skill of undergraduate students in computational work |
| Short Title: | Image analysis applications and self-efficacy of undergraduates in computing |
| Corresponding Author: | Tessa Durham Brooks Doane University UNITED STATES |
| Keywords: | self-efficacy in computing; computational thinking; undergraduate education; education research |
| Abstract: | In many areas of science, the ability to use computers to process, analyze, and visualize large data sets has become essential. The mismatch between the ability to generate large data sets and the computing skill to analyze them is arguably the most striking within the life sciences. The Digital Image and Vision Applications in Science (DIVAS) project describes a scaffolded series of interventions implemented over the span of a year to build the coding and computing skill of undergraduate students majoring primarily in the natural sciences. The program is designed as a community of practice, providing support within a network of learners. The program focus, images as data, provides a compelling "hook" for participating scholars. Scholars begin the program with a one-credit spring semester seminar where they are exposed to image analysis. The program continues in the summer with a one-week, intensive Python and image processing workshop. From there, scholars tackle image analysis problems using a pair programming approach and finish the summer with independent research. Finally, scholars participate in a follow-up seminar the following spring and help onramp the next cohort of incoming scholars. We observed promising growth in participant self-efficacy in computing that was maintained throughout the project as well as significant growth in key computational skills. DIVAS program funding was able to support seventeen DIVAS over three years, with 76% of DIVAS scholars identifying as women and 14% of scholars being members of an underrepresented minority group. Most scholars (82%) entered the program as freshmen, with 89% of DIVAS scholars retained for the duration of the program and 100% of scholars remaining a STEM major one year after completing the program. The outcomes of the DIVAS project support the efficacy of building computational skill through repeated exposure of scholars to relevant applications over an extended period within a community of practice. |
| Order of Authors: | Tessa Durham Brooks |
|  | Raychelle Burks |
|  | Erin Doyle |
|  | Mark Meysenburg |
|  | Timothy Frey |
| Additional Information: |  |
| Question | Response |
| Financial Disclosure  Enter a financial disclosure statement that describes the sources of funding for the work included in this submission. Review | TDB, RB, MM - 1608754 - National Science Foundation - [https://www.nsf.gov/awardsearch/showAward?AWD_ID=1608754&HistoricalAwards=fa](http://www.nsf.gov/awardsearch/showAward?AWD_ID=1608754&HistoricalAwards=fa) lse  TDB, ED - 1557417 - National Science Foundation - |

*Powered by Editorial Manager® and ProduXion Manager® from Aries Systems Corporation*

| the [submission guidelines](http://journals.plos.org/plosone/s/submission-guidelines#loc-financial-disclosure-statement) for detailed requirements. View published research articles from [PLOS ONE](http://journals.plos.org/plosone/) for specific examples.  This statement is required for submission and will appear in the published article if the submission is accepted. Please make sure it is accurate. | [https://www.nsf.gov/awardsearch/showAward?AWD_ID=1557417&HistoricalAwards=fa](http://www.nsf.gov/awardsearch/showAward?AWD_ID=1557417&HistoricalAwards=fa) lse  RB - BH-0018 - The Welch Foundation - [https://www.welch1.org/](http://www.welch1.org/)  The funders had no role in study design, data collection and analysis, decision to publish, or preparation of the manuscript. |
| --- | --- |
| Unfunded studies  Enter: The author(s) received no specific funding for this work.  Funded studies  Enter a statement with the following details:   - Initials of the authors who received each award - Grant numbers awarded to each author - The full name of each funder - URL of each funder website - Did the sponsors or funders play any role in the study design, data collection and analysis, decision to publish, or preparation of the manuscript? - NO - Include this sentence at the end of your statement: The funders had no role in study design, data collection and analysis, decision to publish, or preparation of the manuscript. - YES - Specify the role(s) played. |  |
| * typeset |  |
| Competing Interests  Use the instructions below to enter a competing interest statement for this submission. On behalf of all authors, disclose any [competing interests](http://journals.plos.org/plosone/s/competing-interests) that could be perceived to bias this work—acknowledging all financial support and any other relevant financial or non- financial competing interests.  This statement will appear in the published article if the submission is accepted. Please make sure it is accurate. View published research articles from [PLOS ONE](http://journals.plos.org/plosone/) for specific examples. | The authors have declared that no competing interests exist. |

*Powered by Editorial Manager® and ProduXion Manager® from Aries Systems Corporation*

| NO authors have competing interests  Enter: The authors have declared that no competing interests exist.  Authors with competing interests  Enter competing interest details beginning with this statement:  I have read the journal's policy and the authors of this manuscript have the following competing interests: [insert competing interests here] |  |
| --- | --- |
| * typeset |  |
| Ethics Statement  Enter an ethics statement for this submission. This statement is required if the study involved:   - Human participants - Human specimens or tissue - Vertebrate animals or cephalopods - Vertebrate embryos or tissues - Field research   Write "N/A" if the submission does not require an ethics statement.  General guidance is provided below. Consult the [submission guidelines](http://journals.plos.org/plosone/s/submission-guidelines#loc-guidelines-for-specific-study-types) for detailed instructions. Make sure that all information entered here is included in the Methods section of the manuscript. | The Doane University IRB approved the study under proposal S17 001 DC IRB HS via written approval. |

*Powered by Editorial Manager® and ProduXion Manager® from Aries Systems Corporation*

| Format for specific study types  Human Subject Research (involving human participants and/or tissue)   - Give the name of the institutional review board or ethics committee that approved the study - Include the approval number and/or a statement indicating approval of this research - Indicate the form of consent obtained (written/oral) or the reason that consent was not obtained (e.g. the data were analyzed anonymously)   Animal Research (involving vertebrate animals, embryos or tissues)   - Provide the name of the Institutional Animal Care and Use Committee (IACUC) or other relevant ethics board that reviewed the study protocol, and indicate whether they approved this research or granted a formal waiver of ethical approval - Include an approval number if one was obtained - If the study involved non-human primates, add additional details about animal welfare and steps taken to ameliorate suffering - If anesthesia, euthanasia, or any kind of animal sacrifice is part of the study, include briefly which substances and/or methods were applied   Field Research  Include the following details if this study involves the collection of plant, animal, or other materials from a natural setting:   - Field permit number - Name of the institution or relevant body that granted permission |  |
| --- | --- |
|  |  |
| Data Availability  Authors are required to make all data underlying the findings described fully available, without restriction, and from the time of publication. PLOS allows rare exceptions to address legal and ethical concerns. See the [PLOS Data Policy](http://journals.plos.org/plosone/s/data-availability) and [FAQ](http://journals.plos.org/plosone/s/data-availability#loc-faqs-for-data-policy) for detailed information. | Yes - all data are fully available without restriction |

*Powered by Editorial Manager® and ProduXion Manager® from Aries Systems Corporation*

| A Data Availability Statement describing where the data can be found is required at submission. Your answers to this question constitute the Data Availability Statement and will be published in the article, if accepted. |  |
| --- | --- |
| Important: Stating ‘data available on request from the author’ is not sufficient. If your data are only available upon request, select ‘No’ for the first question and explain your exceptional situation in the text box. |  |
| Do the authors confirm that all data underlying the findings described in their manuscript are fully available without restriction? |  |
| Describe where the data may be found in full sentences. If you are copying our sample text, replace any instances of XXX with the appropriate details.   - If the data are held or will be held in a public repository, include URLs, accession numbers or DOIs. If this information will only be available after acceptance, indicate this by ticking the box below. For example: All XXX files are available from the XXX database (accession number(s) XXX, XXX.). - If the data are all contained within the manuscript and/or Supporting Information files, enter the following: All relevant data are within the manuscript and its Supporting Information files. - If neither of these applies but you are able to provide details of access elsewhere, with or without limitations, please do so. For example:   Data cannot be shared publicly because of [XXX]. Data are available from the XXX Institutional Data Access / Ethics Committee (contact via XXX) for researchers who meet the criteria for access to confidential data.  The data underlying the results presented in the study are available from (include the name of the third party | All relevant data are within the manuscript and its Supporting Information files. |

*Powered by Editorial Manager® and ProduXion Manager® from Aries Systems Corporation*

| and contact information or URL).   - This text is appropriate if the data are owned by a third party and authors do not have permission to share the data.   * typeset |  |
| --- | --- |
| Additional data availability information: |  |

*Powered by Editorial Manager® and ProduXion Manager® from Aries Systems Corporation*

[Click here to access/download;Manuscript;msfile-1.docx](https://www.editorialmanager.com/pone/download.aspx?id=27681744&guid=a538f4cc-740b-4a09-8cf5-7797be26a59c&scheme=1)

1

2

3

## 4 Digital Imaging and Vision Analysis in Science Project improves the self-efficacy and

5 skill of undergraduate students in computational work

## 6 Short title: Image analysis applications and self-efficacy of undergraduates in computing

7

## 8 Tessa Durham Brooks^1*^, Raychelle Burks^2^, Erin Doyle^1^, Mark Meysenburg^3^, and Tim

9 Frey^4^.

10

## 11 Departments of ^1^Biology, ^3^Computer Science, and ^4^Education, Doane University, Crete,

12 NE, 68333; ^2^Department of Chemistry, American University, Washington, DC, 20016

13

14

15

## 16 *Corresponding author

17 Email: [tessa.durhambrooks@doane.edu](mailto:tessa.durhambrooks@doane.edu) (TDB)

18

19

20

21

22

23

24

25

26	**Abstract**

27

28 In many areas of science, the ability to use computers to process, analyze, and visualize large
29 data sets has become essential. The mismatch between the ability to generate large data sets
30 and the computing skill to analyze them is arguably the most striking within the life sciences.
31 The Digital Image and Vision Applications in Science (DIVAS) project describes a scaffolded
32 series of interventions implemented over the span of a year to build the coding and computing
33 skill of undergraduate students majoring primarily in the natural sciences. The program is
34 designed as a community of practice, providing support within a network of learners. The
35 program focus, images as data, provides a compelling ‘hook’ for participating scholars. Scholars
36 begin the program with a one-credit spring semester seminar where they are exposed to image
37 analysis. The program continues in the summer with a one-week, intensive Python and image
38 processing workshop. From there, scholars tackle image analysis problems using a pair
39 programming approach and finish the summer with independent research. Finally, scholars
40 participate in a follow-up seminar the following spring and help onramp the next cohort of
41 incoming scholars. We observed promising growth in participant self-efficacy in computing that
42 was maintained throughout the project as well as significant growth in key computational skills.
43 DIVAS program funding was able to support seventeen DIVAS over three years, with 76% of
44 DIVAS scholars identifying as women and 14% of scholars being members of an
45 underrepresented minority group. Most scholars (82%) entered the program as freshmen, with
46 89% of DIVAS scholars retained for the duration of the program and 100% of scholars
47 remaining a STEM major one year after completing the program. The outcomes of the DIVAS
48 project support the efficacy of building computational skill through repeated exposure of
49 scholars to relevant applications over an extended period within a community of practice. 50

### 51 Introduction

52

53 Science, technology, engineering, and mathematics (STEM) professions, even those not
54 traditionally steeped in quantitative models and data analysis, increasingly require
55 computational competence [[1]](https://paperpile.com/c/GCFfyr/U0fD). In particular, the natural sciences have experienced significant
56 increases in the amount of data generated by increased computing power, cheaper and more
57 rapid sequencing technologies, and the rise of interdisciplinary fields such as personalized
58 medicine, phenomics, digital agriculture, and climate science. Computation has become so
59 ubiquitous and necessary across the natural and physical sciences that it has been referred to
60 as the “third pillar of the scientific method,” along with theory and experimentation [[2]](https://paperpile.com/c/GCFfyr/vazY). A career
61 in the natural sciences increasingly requires that professionals are comfortable with basic
62 computational skills and quantitative analysis [[3–5]](https://paperpile.com/c/GCFfyr/fUJR%2BLpP2%2BaKSn). Beyond this, modern scientific exploration
63 may require the design of new software by developers with both specific content knowledge and
64 computational skills. As a potential "end user", a biologist, chemist, physicist, etc. has the
65 content knowledge, but may need computational skills training [[6,7]](https://paperpile.com/c/GCFfyr/4MF1%2Bpbxy). Across the broad range of
66 STEM disciplines, too few students are being trained in computational and quantitative skills
67 that would enable them to develop useful software. In particular, undergraduate students in the
68 life sciences may be resistant to developing quantitative or computational skills due to previous
69 negative experiences or a perception that they “aren’t good at” mathematics or computers [[8]](https://paperpile.com/c/GCFfyr/guYF).
70 The result of these factors is a mismatch between the skills needed for success in research or
71 industry positions and the skills possessed by graduates and young professionals starting these
72 positions.

73

74 To address this mismatch, we conceived of the Digital Imaging and Vision Applications in
75 Science (DIVAS) Project. This year-long program was designed as a guided ‘onramp’ to
76 develop computational skills within a community of practice that would contribute to participants’
77 STEM career success. The overall goal of the DIVAS project is to develop, utilize, and test
78 interventions that will engage and train STEM undergraduate students in computing - especially
79 students that do not traditionally participate in computer science curriculum. DIVAS
80 interventions present students with visually-appealing image-based problems relevant to the
81 disciplines they are majoring in, thereby making the skills we aim to develop eminently practical.
82 Importantly, it is relatively easy to capture images with high spatial, temporal, and spectral
83 resolution, with images being increasingly used as data in scientific, clinical, and engineering
84 settings [[9–12]](https://paperpile.com/c/GCFfyr/M19r%2BBb34%2BoXJC%2BSl4h). While images are relatively easy to obtain, extracting useful information from
85 them commonly presents technical barriers that lead to processing bottlenecks. Although the
86 collection of large datasets has become rather commonplace, scientists of various career
87 stages may lack the computational skills to analyze these data independently or may have
88 limited access to productive collaborations with computer scientists or other specialists. Early
89 introduction to computational approaches, along with frequent practice, enables a person new to
90 computing to take advantage of training resources to develop critical skills and to form effective
91 collaborations [[13–15]](https://paperpile.com/c/GCFfyr/ufYw%2BhiKM%2Bx61G). Studies of computer science courses that present instructional concepts
92 in the context of digital images, videos, or music - i.e. “media computation” [[16]](https://paperpile.com/c/GCFfyr/M2go) - improves
93 retention of women and non-computer science majors in these courses [[13,15,17,18]](https://paperpile.com/c/GCFfyr/ufYw%2Bx61G%2B0k9Q%2BAwLH). 94
95 Just as computation-in-context supports student gains, so do communities of practice and
96 learning communities. Both types of communities, which can be quite distinct depending on their
97 specific model [[19]](https://paperpile.com/c/GCFfyr/waio), are often used interchangeably to describe a community for sharing,
98 developing, and/or maintaining knowledge, skills, and practices within which membership
99 ranges from novices to seasoned experts. For students, participation in such communities has
100 been shown to boost academic performance, self-efficacy, sense of belonging, STEM identity,
101 retention, and graduation rates [[20–23]](https://paperpile.com/c/GCFfyr/hiBD%2B27Tr%2Bgsd2%2BtdGl). In the DIVAS Project, cohorts of novices work side-by-
102 side with faculty mentors, and their more experienced student peers, to themselves become
103 more advanced practitioners via legitimate peripheral participation [[24]](https://paperpile.com/c/GCFfyr/52b6). Importantly, the DIVAS
104 Project models the reality of the modern computational work environment, which is soundly a

team-based endeavor. This counters the stereotype that such work is largely solitary.

107 The general hypothesis of the DIVAS Project is that gradual, scaffolded exposure to - and
108 practice with - computational tools, centered on accessible and relevant applications, and
109 implemented in both simulated and authentic supportive professional environments, will impact
110 student self-efficacy, computational competency, and career path interest. We have taken the
111 approach of emphasizing growth in self-efficacy toward computing as the first necessary
112 indicator of growth in computational skill [[25–27]](https://paperpile.com/c/GCFfyr/glYw%2BPKxV%2BBktK). We also posit that as participants become
113 more familiar with computational tools, they will additionally show more interest in career paths
114 that would utilize said tools. Though our pilot program was restricted in size, its positive impact
115 on participants suggests that DIVAS program elements are well-suited to our broader goals of
116 fostering computation skills within a community of practice. We describe our approach here both
117 as a guide and an invitation. We hope to form new DIVAS partnerships to broaden the DIVAS
118 community and enable additional study on the efficacy of the approach we have taken. 119

### 120 DIVAS Program Elements

121 To explore our hypothesis, a pathway of interventions was designed that comprise our
122 programmatic ‘onramp’ (Fig 1). Each cohort of DIVAS scholars was introduced to our
123 community of practice via a one-credit, spring semester seminar (DIVAS Seminar I) and
124 engagement with the DIVAS Slack team. Work continued in the summer with a week-long
125 coding workshop, followed by a four-week long paired-programming session that allows DIVAS
126 scholars to put their recently acquired skills to use. DIVAS Scholars can participate in an
127 additional three weeks of research with DIVAS faculty to conclude their summer activities. In the
128 fall semester, DIVAS Scholars returning to research - or starting new computing projects -
129 continue engaging with community members using our Slack team. During the following spring,
130 the cohort takes DIVAS Seminar II. As with other team science endeavors, the DIVAS
131 community is offline and online, with Slack and Zoom playing significant roles in communication,
132 project management, co-working sessions, team meetings, etc. In the sections that follow, the
133 basic design of each intervention is detailed and intervention resources can be found at the

DIVAS Program Resources website [[28]](https://paperpile.com/c/GCFfyr/OCmr).

136 Fig 1: Interventions comprising the computational ‘onramp’ of the DIVAS program. 137
138 ***DIVAS Seminar I and II.***	DIVAS Seminar I and II are both one-credit seminars offered in the
139 spring semester. DIVAS Seminar I is offered to new scholars before the summer coding
140 workshop and projects. DIVAS Seminar II is offered to scholars the spring after they have
141 completed the summer interventions. DIVAS Seminar I is designed to introduce students to
142 images as data and basic coding concepts, as well as allow them to meet professionals who
143 use coding in their everyday work. Students complete a photo journal project where they identify
144 a question or problem of interest, collect a series of images to address that question or problem,
145 then use ImageJ to conduct simple image processing. In DIVAS Seminar II, students clean-up
146 and annotate Python code written the previous summer. They also work with the instructor to
147 make edits and improvements to the coding workshop as needed. Finally, students learn about
148 and gain some familiarity with parallelization and grid computing. Course syllabi and sample
149 resources for each course are available at the DIVAS Program Resources website [[28]](https://paperpile.com/c/GCFfyr/OCmr). 150
151 ***Coding	Workshop.***	Short courses, such as those run by The Carpentries, have become a
152 popular way to build coding and data analysis skills [[29]](https://paperpile.com/c/GCFfyr/fb6s). On average, participants report
153 increased self-efficacy in coding and coding skills, based on pre- and post-workshop surveys
154 and on longitudinal surveys [[29,30]](https://paperpile.com/c/GCFfyr/fb6s%2BTaWX). However, workshops like those offered through The
155 Carpentries are not targeted towards, nor significantly attended by, undergraduate students
156 [[29]](https://paperpile.com/c/GCFfyr/fb6s). We designed a one-week coding workshop that includes two days of basic coding in
157 Python and three days of image processing using OpenCV libraries. The two-day introduction to
158 Python was modeled on an existing Carpentries workshop and can be found at GitHub [[31]](https://paperpile.com/c/GCFfyr/BZei). The
159 overall design of the three-day image processing workshop was informed by Adrian
160 Rosebrook’s 2016 book on the topic [[32]](https://paperpile.com/c/GCFfyr/6fnX). To keep students engaged with Python basics,
161 examples used during this section of the workshop were tailored toward image processing
162 projects. Students were also presented with two authentic and “solvable” research problems at
163 the beginning of the image processing portion of the workshop. For the first problem,
164 participants were asked to count bacterial colonies on a plate image. For the second,
165 participants were asked to track the progress of an acid-base titration captured on video. Our
166 workshop design provides students an opportunity to immediately apply their recently acquired
167 Python skills to write code to perform analysis tasks to address these two authentic problems.
168 The image processing portion of the workshop was adopted by The Carpentries in 2019 [[30,33]](https://paperpile.com/c/GCFfyr/TaWX%2B9jH3).
169 At the same time, the image processing operations were translated into Scikit-image, which is
170 much easier to install and implement across a wide range of hardware, software, and network
171 environments. Workshop materials are available at its Data Carpentry site [[30]](https://paperpile.com/c/GCFfyr/TaWX). 172
173 ***Pair Programming Projects.***	Pair programming is a practice used in the software development
174 industry in which two programmers work together, with one person assuming the role of the
175 “driver” who writes the code, and the other taking the role of “observer” who reviews the code
176 and makes suggestions. In introductory computer science courses, the use of pair programming
177 results in higher quality code, increased student enjoyment, improved pass rates for courses,
178 and improved retention in computer science majors for both men and women [[17,34–36]](https://paperpile.com/c/GCFfyr/0k9Q%2BNl97%2BZ1g4%2BwXxD). Also,
179 pair programming has been shown to increase the confidence of women in the programming
180 solutions they produce [[34]***.***](https://paperpile.com/c/GCFfyr/Nl97) We designed the DIVAS program so that participants would
181 transition from the coding workshop to pair programming work, applying knowledge gained in
182 the workshop to the completion of two consecutive two-week pair programming projects. Each
183 year, one project was morphometric in nature while the other was colorimetric. Image data sets
184 were found from public repositories or from the research of the faculty team. The project was
185 presented by a faculty member at the beginning of each project. DIVAS Scholars were randomly
186 divided into pairs. For pairs composed of students at different institutions, pair programming was
187 conducted virtually using Zoom. This arrangement allowed us to explore the feasibility of a fully
188 online program. A significant amount of project management was done via the DIVAS Slack
189 team. Each day, pairs met for a stand-up (brief 5-10 minute) meeting where progress and next
190 steps were reported. Issues were also shared and discussed. Pairs worked on code for the
191 remainder of the day. A formal code review was conducted each week by the DIVAS community
192 of practice, with community members joining both in-person and virtually. All participants were
193 to have copied and annotated the code of the other teams prior to the review. Progress and
194 issues were discussed. As a group, major goals for the following week or final items to wrap up
195 the project were identified. An example of a pair programming project can be found at the
196 DIVAS Program Resources website [[28]](https://paperpile.com/c/GCFfyr/OCmr). 197
198 ***Independent	Research.***	In year one, scholars were required to conduct 3-4 weeks of
199 independent research after completing pair programming. Projects were based off of the
200 existing research of the faculty team as well as were informed by student interest (Table 1).
201 Students generally worked independently, but met with their faculty advisor for daily check-ins
202 and to troubleshoot any problems that arose. Participating in DIVAS research was optional in
203 years two and three to better accommodate student schedules, e.g. REU participation, study

abroad, etc.

**Table 1.** Example Pair Programming and Research Projects

1. Detection of breaks in veterinary x-ray images

2. Detection and quantification of standards printed onto a

solid surface

3. Calculating the endpoint of a titration from a movie of the reaction

4. Counting plaques on an agar plate

5. Quantifying chemotaxis of bacteria toward potential attractants

6. Measuring growth of maize seedlings over time

7. Automatically analyzing and solving images of printed Sudoku problems

8. Improving script performance by converting code from python to C to improve script performance

207 Within the DIVAS Project framework, several questions were explored: 1) How do program
208 interventions impact participant self-efficacy toward computation? 2) How do program
209 interventions impact participant career interest? 3) How do program interventions and their
210 ability to demonstrate effective computational thinking?
211 The overall objectives of the DIVAS project are to:
212 1. Explore the effectiveness of coding workshops on student attitudes toward computation
213 and their ability to demonstrate effective computational thinking.
214 2. Measure impacts of paired programming projects, independent research, and
215 professional development seminars on self-efficacy and ability to apply computational
216 skills.
217 3. Investigate the impact of curricular and co-curricular interventions in computation on
218 student preferred and actual career path. 219

### 220 Materials and Methods

221

222 ***Study Context.*** Each of the three years of the study, the DIVAS program was advertised using
223 flyers (digital and paper), online and social media posts, and visits by faculty and existing
224 scholars to classes that are generally enrolled by freshman and sophomore natural science
225 majors. Up to six scholars were selected each year. Every effort was made to include each
226 student who completed an application in the DIVAS program. A total of 17 scholars were
227 selected to complete all program interventions (Table 2). Scholars were 76% women and 14%
228 underrepresented minority (URM; African Americans, American Indians including Native
229 Alaskans, Hispanics and Native Pacific Islanders). An additional 17 faculty, staff, and students

participated in the one-week coding workshop and completed pre- and post-assessments.

232 Scholars’ majors were biomedical engineering, biology, biochemistry, computer science, health
233 and human performance, health and society, chemistry, and bioinformatics. STEM major
234 retention was 100% at one year after the DIVAS II Seminar, with 89% of DIVAS Scholars
235 retaining a STEM major for the duration of the program. Participants provided informed consent
236 to provide self-efficacy and career path data by completing and submitting an electronic survey
237 administered using Qualtrics software (Qualtrics, Provo, UT). Participants also provided
238 informed consent to complete computational thinking prompts and to submit code generated for
239 analysis, which was scored by project researchers. Doane University Institutional Review Board
240 (IRB) approved the study. 241

**Table 2.** Participant overview. Participants who completed assessments, with % women in parenthesis.

|  | **Year 1** | **Year 2** | **Year 3** | **Total** |
| --- | --- | --- | --- | --- |
| **DIVA Scholars** | 6 (66%) | 6 (83%) | 5 (80%) | 17 (76%) |
| **Coding**  **Workshops** | 14 (50%) | 10 (71%) | 10 (58%) | 34 (59%) |

242

243 ***Self -efficacy and Career Path Assessment.***	A Qualtrics survey was used to measure
244 perceived self-efficacy in computing and intention to pursue a career path involving computing.
245 This survey, titled ‘DIVAS Career Path and Self-Efficacy’, is based on two previously-designed
246 and validated surveys [[37,38]](https://paperpile.com/c/GCFfyr/AE4W%2BoDRX). Survey questions ask participants to score their general
247 knowledge of computational thinking and ability to use computational tools to solve problems; to
248 indicate how much they know about careers using computer science applications, programming
249 or computational thinking; and to assess how familiar they are with how to find information about
250 computationally-related careers. Twelve questions related to self-efficacy are answered as a
251 user-inputted number on a 100-point scale, with higher values representing more self-efficacy
252 for a particular item. Seven questions related to career paths include response choices on a
253 four- or five-point Likert-type scale. Participants took the survey before and after the major
254 interventions in the project. If the participant had previously completed the survey after an
255 intervention, this score was used as the pre-survey for a subsequent intervention. A PDF of the
256 survey can be found under ‘Assessment Tools’ at the DIVAS Program Resources website [[28]](https://paperpile.com/c/GCFfyr/OCmr). 257
258 ***Computational Thinking Assessment.***	A rubric was designed and iteratively revised to
259 measure computational thinking based on definitions from the International Society for
260 Technology in Education (ISTE) and Computer Science Teachers Association (CSTA),
261 Carnegie Mellon, Google, and Harvard [[39–42]](https://paperpile.com/c/GCFfyr/FJ1j%2BgxzC%2BNefq%2B570i). Our computational thinking (CT) rubric was
262 organized into the first four phases of the RADIS (Recognize / Analyze / Design / Implement /
263 Support) framework [[43]](https://paperpile.com/c/GCFfyr/BSMR). The Recognize section measures how well the problem is understood
264 and one’s ability to gather the data needed to solve the problem. The Analyze section measures
265 the ability of the participant to understand the options available to solve the given problem. This
266 section also measures the ability of the participant to use abstraction, modeling/representation,
267 and decomposition to design a solution to a problem. The Design section measures the
268 participant’s ability to design an effective algorithmic procedure to solve the problem. It includes
269 the participant’s ability to use sequence, selection, and iteration. The Implementation section
270 addresses the ability of the participant to transform the algorithm into working code to solve a
271 given problem. It also addresses the evidence that is used, reused, and remixed from previous
272 projects or other sources. Finally, the Implement section assesses any testing or debugging that
273 was used to improve the code. The original CT rubric was scored on a three-point scale;
274 Proficient (3), Progressing (2), or Novice (1). Subsequent iterations included five levels, first
275 from 0 to 4, then from 1 to 5. The additional levels were added to better accommodate the types
276 of variation we were seeing in the scored artifacts. The expanded scale was adjusted to start
277 with ‘1’ (indicating that something was attempted) from ‘0’ (indicating that nothing was
278 attempted) to make statistical analysis more interpretable. Scales were standardized in the
279 analysis so that group differences were comparable from year to year. The internal reliability of
280 the first version and final versions of the instrument was high overall (Cronbach’s alpha of 0.94-
281 0.95). Interrater reliability was determined to be 76% using a set of seven artifacts scored by
282 three raters. The reliability of each section for both the first and final versions ranged from a
283 Cronbach’s alpha of 0.80 for the ‘Implement’ section to a Cronbach’s alpha of 0.97 for the
284 ‘Design’ section. The iterations of the CT rubric are available under ‘Assessment Tools’ at the

DIVAS Program Resources website [[28]](https://paperpile.com/c/GCFfyr/OCmr).

287 To assess CT ability before any formal instruction in coding, participants were given a handout
288 that described a hypothetical cup stacking robot that could be given simple instructions to
289 achieve different configurations of cups. The exercise was adapted from the Hour of Code
290 lesson “Programming Unplugged: My Robotic Friends” [[44]](https://paperpile.com/c/GCFfyr/LhSs). Participants were asked to create a
291 series of commands to achieve a particular cup stacking arrangement. After writing their initial
292 set of commands, participants were asked to simplify their ‘code’, possibly by writing one or
293 more new commands. A different cup-stacking prompt was used after the DIVAS Seminar I.
294 After each subsequent intervention, the code developed in each one was used to assess CT
295 ability. The cup stacking prompts are under ‘Assessment Tools’ at the DIVAS Program

Resources website [[28]](https://paperpile.com/c/GCFfyr/OCmr).

298 ***Data Analysis.*** To investigate changes in student self-efficacy and career interest, scores within
299 each category were summed to determine a composite score for each individual. A paired-
300 samples t-test was performed (alpha = 0.05) to determine if composite scores before and after a
301 given intervention were significant. For significant changes, the effect size was determined by
302 calculating Cohen's d. The change in CT scores within a year was determined by calculating a
303 total score for an individual artifact from each rater and determining the median value. A paired-
304 samples t-test was performed to determine if CT scored had changed after an intervention.
305 Subscores within each of the areas of the rubric (Recognize, Analyze, Design, Implement) were
306 also calculated and evaluated using a paired-samples t-test to determine whether significant
307 changes within each area were observed. Effect sizes for significant differences were described
308 by calculating Cohen’s d. 309

### 310 Results and Discussion

311

312 Up to six scholars were selected each year of this three-year project, with a total of 17 scholars
313 participating overall. Participant limits were due to budget constraints. Most scholars were
314 women (76%) and 14% were a member of an URM. Most scholars (82%) were in their first year
315 of college when starting the program. In addition to the DIVAS scholars, seventeen individuals
316 participated in the coding workshop, 47% of whom were members of an URM group (Table 2).
317 Scholars start the program with a one-credit seminar in the spring and end the program with a
318 one-credit seminar the following spring, thereby participating in the full DIVAS pipeline as

presented in Fig 1 (above).

321 The project measured the impact of DIVAS interventions on participant self-efficacy in using
322 computation to solve problems, participants’ attitudes towards computation, and participant
323 awareness/interest in computing careers using established instruments (see Materials and
324 Methods). For all of the interventions of the DIVAS pipeline, self-efficacy toward computing and
325 intended career path was assessed. Computational thinking was assessed in all of the
326 interventions except DIVAS Seminar II. Pre- and post-assessments for each intervention were
327 completed by participants. The post-test data from the prior intervention was used as a baseline
328 for the next intervention in the pipeline. Overall, every intervention was found to have a positive
329 effect on one or more measures (self-efficacy, career interest, computational thinking) for at
330 least one of the program years. In the proceeding sections, we discuss the assessment results
331 for each intervention and conclude with overall program impacts. 332

#### 333 Self -efficacy and Career path data by intervention

334

335 ***DIVAS Seminar I.***	As described in DIVAS Program Elements, this seminar is the scholar’s entry
336 point onto the DIVAS programmatic onramp. We saw significant gains in self-efficacy or career
337 path each year of the program and in aggregate (Table 3). 338

**Table 3.** Self-efficacy and career path gains in DIVAS Seminar I. Effect sizes (Cohen’s-D) for each year of the program and the three years combined (Comb.) is shown. *, p < 0.5.

|  | **Year 1** | **Year 2** | **Year 3** | **Comb.** |
| --- | --- | --- | --- | --- |
| **Self -efficacy** | 2.38* | ns | 0.74* | 0.35* |
| **Career path** | ns | 1.38* | ns | 0.63* |

339

340 An additional source of self-efficacy information came from the voluntary completion of an IDEA
341 Student Ratings of Instruction system survey [[45]](https://paperpile.com/c/GCFfyr/To6r), which is conducted at Doane University at the
342 end of each course and that we utilized in the DIVAS project. We analyzed self-reported
343 learning gains in the IDEA-defined learning objectives for the eleven scholars who completed
344 the survey (year 1 = 5, year 2 = 4, year 3 = 2). We found that scholars self-reported strong gains
345 in the objectives “Acquiring skills in working with others as a member of a team” and “Learning
346 appropriate methods for collecting, analyzing, and interpreting numerical information.” The three
347 cohorts rated both objectives at an average score of 4.45 out of 5 points. The Doane
348 institutional average over the period of this project on these learning goals are 3.72 and 3.56,
349 respectively. Overall, DIVAS Seminar I was effective in improving the self-efficacy of Scholars
350 toward computing, and positively influencing their intended career path. Observationally, the
351 seminar was important in building rapport and a shared experience between all members
352 (faculty and students) in the community of practice. In year three, the DIVAS cohort completed
353 their photo diary project in tandem with 200-level graphic design students (Fig 2). This
354 experience was one of several opportunities that the seminar provided to anchor image

collection and analysis in relevant ways.

357 Fig 2: A collaborative photo journal project. A DIVAS scholar used ImageJ to analyze images of
358 a healing wound (top) while a design student created a composition depicting the healing
359 process (bottom). 360
361 ***Coding workshop.***	Modeled after existing Carpentries workshops, the five-day DIVAS
362 workshop included two days of basic Python, Bash shell, and Git skills and a custom three-day
363 workshop on basic computer vision topics using the OpenCV library for Python. The workshop
364 was built around participants solving authentic research challenges within a community of
365 practice focused on computation skills development (see ’DIVAS Program Elements’). We
366 gathered self-efficacy and career path data pre- and post-workshop. We saw a significant
367 improvement in self-efficacy in aggregate over the three years (Cohen’s-d = 0.57, p < 0.01).
368 There were no significant changes in intended career path over the three-year period. However,
369 we did observe an increase in the standard deviation of the mean score. In looking at individual
370 responses, this increase in standard deviation seems to indicate that scholars became more
371 extreme at either end in their interest in incorporating computational skills in their future careers
372 after this intervention. We did not find this concerning since this divergence in interest was

paired with a significant increase in self-efficacy.

375 At the end of each day of the workshop, we also asked participants to rate the percentage of the
376 day’s material they felt they had mastered. Data was compiled for all participants, including
377 those who were not DIVAS scholars. We found a high average perceived mastery for the
378 Python/Bash/git portion of the workshop, and then a drop for the first two days of the computer
379 vision portion (Table 4). We believe this is due to the increased complexity in the subject matter.
380 By the third day, this metric rose as participants were able to use their newfound skills to
381 complete the challenge questions successfully. 382

**Table 4.** Participant responses to the question ‘What percentage of the day’s material do you feel you have mastered?’ for each day of the Coding Workshop.

|  | **Day 1** | **Day 2** | **Day 3** |
| --- | --- | --- | --- |
| **Python Intro** | 75.9% | 76.3% | --- |
| **Image Processing** | 63.3% | 63.0% | 72.2% |

383

384 We found the coding workshop format to be effective as it immersed scholars in an enriching
385 skill development environment. Though coding training was intensive, the participants’ self-
386 reported improvements in mastery support the observation that scholars see tangible benefits
387 from their persistence. The workshop also provided two cycles of challenge, learn, and achieve
388 - in the spirit of Challenge Based Learning [[46]](https://paperpile.com/c/GCFfyr/ypra) - to provide participants multiple opportunities to
389 struggle with new concepts and see the payoff. 390
391 ***Pair -Programming Projects and Research.***	Following the coding workshop, scholars
392 employed pair-programming to solve a colorimetric and a morphometric image analysis problem
393 (Fig 3). For each problem, each pair developed code to extract relevant data from the images,
394 analyze and present the data appropriately, and validate their results. To promote a community
395 of practice, scholars participated in daily “stand-up” meetings where they gave brief progress
396 reports and set goals for the day. Scholars participated in weekly guided code review sessions
397 where they presented their code and provided critical feedback (written and verbal) on each
398 pair’s code. The process was repeated with a second project with different partners in the

following two weeks.

401 Following pair programming work, scholars had the opportunity to complete three or more
402 weeks of independent research (required in Year 1, optional in Years 2 and 3). Scholars either
403 chose to work on existing projects, or design their own, within a faculty mentored research
404 group. Examples of scholars’ projects included locating breaks in veterinary x-ray images,
405 ingesting and solving printed Sudoku problems, greatly improving program performance by
406 translating Python scripts into parallelized C++ code, and measuring chemotaxis of bacteria

toward potential attractants (Table 1, above).

409 Fig 3: Example pair programming project in which students aimed to count the dead (straight)

worms in a series of images. The unprocessed image is from [[47]](https://paperpile.com/c/GCFfyr/oi2O).

412 We collected self-efficacy and career path data upon the conclusion of summer research, if the
413 scholar participated, or at the conclusion of pair-programming projects for those students not
414 participating in research. Although self-efficacy moved in a positive direction, we did not see
415 significant gains in self-efficacy or career path at the end of summer activities. This was not
416 surprising because students’ self-efficacy was already high and near the ceiling of the
417 instrument, on average, following the coding workshop (Fig. 4). However, given that students
418 were asked to solve a number of challenging problems largely independently, we see the
419 maintenance of self-efficacy throughout this programmatic period as significant.
421 Fig 4: Average self-efficacy scores after DIVAS pipeline interventions for the three years of the

project. Pre = pre-intervention score, PP = pair programming. *, p < 0.05; **, p < 0.01

424 Observationally, the pair-programming and summer research projects were where scholars truly
425 experienced the team-based environment of computational work. They learned to leverage each
426 other’s ideas and expertise to develop approaches to solving a variety of problems. We found
427 that scholars tended to work amongst themselves *before* seeking input from one of the faculty
428 mentors. We considered this both a healthy development of independence and teamwork that
429 reflected the increased confidence scholars gained in their individual and collective skill sets.
430 We also observed cases where one or more scholars would be given special authority by the
431 group. While this was often productive, we also observed that it sometimes contributed to an
432 over/underfunctioning dynamic between pairs. Because of this, we were especially mindful of
433 giving praise for taking risks and highlighting the specific strengths of each project and each
434 scholar separately. We also worked to minimize this over/underfunctioning dynamic when
435 selecting pairs for each project so as to maximize each student’s engagement. 436
437 ***DIVAS Seminar II.***	In the first iteration of DIVAS Seminar II, scholars organized their project
438 code repositories, developed online portfolios regarding their DIVAS experiences, and gave a
439 local conference presentation. Based on student feedback, the next year’s Seminar II was
440 modified to include more challenging academic content. Students learned parallel programming
441 using Python and OpenMPI, creating a “Burning Ship” fractal image [[48]](https://paperpile.com/c/GCFfyr/HvCq) using the Doane
442 University supercomputer, Onyx. The third year, DIVAS Seminar II walked a line between the
443 first two iterations; students worked on cleaning the previous summer's code and keeping the
444 code repository up to date, in addition to helping test the new version of the Image Processing
445 workshop that used the Scikit-Image processing libraries versus OpenCV. DIVAS Seminar II
446 was scheduled at the same time as DIVAS Seminar I each year. This made it easier to promote
447 interactions between the classes. Further developing peer mentorship opportunities, the DIVAS
448 faculty created a “writing center for computing” on campus called the Center for Computing in
449 the Liberal Arts (CCLA)[[49]](https://paperpile.com/c/GCFfyr/VrlC). The center was led by a staff person hired, in part, to serve this
450 role. Upon creation of the CCLA, several DIVAS Scholars signed up to serve as peer mentors,

assisting in the creation of training materials and participating in center activities.

453 In addition to gains in self-efficacy and career path for the first cohort of scholars, a ~~very~~
454 significant gain in career path for second-year scholars was observed (Table 5). Similar to
455 DIVAS Seminar I, responses on the IDEA survey for DIVAS Seminar II were also analyzed for
456 perceived learning gains. Survey data showed that students perceived the largest gains in
457 “Learning appropriate methods for collecting, analyzing, and interpreting numerical information”
458 (4.33 ± 0.52). Scholars also responded positively to the statement, “My background prepared
459 me well for this course's requirements” (4.5 ± 0.55), reflecting the gains in self-efficacy we saw
460 in the survey data. The last year of the seminar occurred during the first wave of the COVID-19
461 pandemic. This resulted in a response rate to the self-efficacy and career path survey that was

too low to report.

**Table 5.** Self-efficacy and career path gains in DIVAS Seminar II. Effect sizes (Cohen’s-D) for Years 1 & 2. *, p < 0.5.

|  | **Year 1** | **Year 2** |
| --- | --- | --- |
| **Self -efficacy** | 1.2* | ns |
| **Career path** | 1.5* | 3.93** |

***Computational thinking.***	CT ability was measured using a rubric designed by the team as

466 described in the Materials and Methods. Participant responses were scored for CT ability for
467 each intervention, except for DIVAS Seminar II, which did not include activities assessable via
468 our rubric. In Year 1, we saw a significant improvement overall CT scores (Cohen’s d = 0.51, p
469 < 0.1) and in the ‘implementation’ criteria for CT skills (Cohen’s d = 0.96, p<0.05) at the end of
470 the coding workshop. We saw significant gains in overall CT scores after pair programming in
471 both Years 2 (Cohen’s d = 2.0, p < 0.05) and 3 (Cohen’s d = 4.1, p < 0.05). There were
472 significant gains in the ‘recognize’, ‘analyze’, and ‘design’ categories in year 2 (Cohen’s d = 1.5-
473 2.1, p < 0.01). There were significant gains in the ‘analyze’, ‘design’, and ‘implement’ categories

474	in year 3 (Cohen’s d = 2.44-4.57, p < 0.05).

475

#### 476 Overall project outcomes and next steps

477

478 Over the three years of the project, scholars experienced significant increases in self-efficacy
479 towards computing from the beginning of Seminar I to the end of summer programming (FIG 4).
480 The most significant gains (p < 0.05) occurred during Seminar I and the coding workshops. The
481 impact of the DIVAS program on scholars’ intended career paths was more subtle. Although
482 scholars did not show significant career path gains from the initial pre-test before Seminar I to
483 the end of summer research (p = 0.12), Seminar I resulted in significant gains in career interest
484 for all years combined, as did Seminar II for Years 1 and 2 (Tables 3 and 5), both of which
485 include explicit career exploration. Scholars were also observed to become ‘warmer’ or ‘colder’
486 to a career utilizing computing as they moved through the program. This effect is apparent in the
487 increased standard deviation in post-intervention career interest scores, which started at ± 2.3
488 after Seminar I, grew to ± 3.02 after the coding workshop, and increased to ± 3.46 after pair-
489 programming/summer research. We see this as an encouraging progression, especially

because scholar self-efficacy grew steadily throughout the program.

492 In a number of ways, DIVAS scholars have persisted in coding and have incorporated skills
493 gained in the DIVAS project into their academic careers, extracurricular activities, and career
494 planning. One scholar majoring in biology declared a minor in software development. A second
495 biology major switched to a bioinformatics major, and two scholars have taken non-required
496 electives that emphasize computational skills. One scholar participated in an external REU
497 program in computational and systems biology, and eight have continued research projects that
498 incorporate coding or computational thinking. Three DIVAS scholars have worked as peer tutors
499 for Doane’s CCLA. One former scholar is pursuing a Ph.D. in chemical biology with a significant
500 computational component to their research and another student who participated in both the

coding workshop and paired programming is pursuing a Ph.D. in complex biosystems.

503 Overall, even given the small sample represented in this study, we see great potential in the
504 DIVAS approach of introducing novice students to computing through a media computing within
505 a community of practice. Thirteen of the 17 DIVAS scholars from the three years of the project
506 (76%) were women and 14% of scholars were members of an URM group, a significantly higher
507 percentage than the total percentage of women and URM group members in the majors
508 represented in the project or in the STEM workforce [[50]](https://paperpile.com/c/GCFfyr/Eafi). The large majority (82%) of scholars
509 entered the program as freshmen. We retained 89% of DIVAS scholars for the duration of the
510 program and retained 100% within a STEM major one year after completing the program. Our
511 findings suggest that the DIVAS approach to computational skills development is a positive
512 experience for students that warrants additional study through the implementation of DIVAS
513 program elements in a broader array of educational contexts. To this end, we hope to form new
514 DIVAS partnerships that will enable an expanded study on the efficacy of the DIVAS approach. 515

### 516 Acknowledgements

517

518 The research described in this manuscript was funded by the National Science Foundation
519 Digital Imaging and Vision Applications in Science Award IUSE-1608754. Students at Doane
520 University participating in the project have also been supported by the National Science
521 Foundation EPSCoR Center for Root and Rhizobiome Innovation Award OIA-1557417. The
522 Welch Foundation (Grant# BH-0018) supported Dr. Burks and students of the Chemistry
523 Department at St. Edward's University. We would like to thank Adam Erck for his leadership in
524 instituting the CCLA and Sarah Zulkoski for her tireless advocacy and support of this project. 525

### 526 Supporting information captions

527

528	Not applicable.

529

530	**References**

531

532 1.	[National Research Council, Division on Engineering and Physical Sciences, Board on](http://paperpile.com/b/GCFfyr/U0fD)
533 [Mathematical Sciences and Their Applications, Committee on the Mathematical Sciences in](http://paperpile.com/b/GCFfyr/U0fD)
534 [2025. The Mathematical Sciences in 2025. National Academies Press; 2013.](http://paperpile.com/b/GCFfyr/U0fD)
535 2.	[Wing JM, Stanzione D. Progress in computational thinking, and expanding the HPC](http://paperpile.com/b/GCFfyr/vazY)
536 [community. Communications of the ACM. 2016. pp. 10–11. doi](http://paperpile.com/b/GCFfyr/vazY)[:10.1145/2933410](http://dx.doi.org/10.1145/2933410)
537 3.	[Woodin T, Celeste Carter V, Fletcher L. Vision and Change in Biology Undergraduate](http://paperpile.com/b/GCFfyr/fUJR)
538 [Education, A Call for Action—Initial Responses. CBE—Life Sciences Education. 2010. pp.](http://paperpile.com/b/GCFfyr/fUJR)

539	[71–73. doi:10.1187/cbe.10-03-0044](http://paperpile.com/b/GCFfyr/fUJR)

540	4.	[Guo P. Why Scientists and Engineers Must Learn Programming. 18 Jul 2013 [cited 20 Jul](http://paperpile.com/b/GCFfyr/LpP2)

541	[2020]. Available:](http://paperpile.com/b/GCFfyr/LpP2) [https://cacm.acm.org/blogs/blog-cacm/166115-why-scientists-and-](https://cacm.acm.org/blogs/blog-cacm/166115-why-scientists-and-engineers-must-learn-programming/fulltext)

542	[engineers-must-learn-programming/fulltext](https://cacm.acm.org/blogs/blog-cacm/166115-why-scientists-and-engineers-must-learn-programming/fulltext)

543	5.	[Janetos AC. A New Biology for the 21st Century. Frontiers in Ecology and the Environment.](http://paperpile.com/b/GCFfyr/aKSn)

544	[2009. pp. 455–455. doi:](http://paperpile.com/b/GCFfyr/aKSn)[10.1890/1540-9295-7.9.455](http://dx.doi.org/10.1890/1540-9295-7.9.455)

545	6.	[Segal J, Morris C. Developing Scientific Software. IEEE Software. 2008. pp. 18–20.](http://paperpile.com/b/GCFfyr/4MF1)

546	[doi:](http://paperpile.com/b/GCFfyr/4MF1)[10.1109/ms.2008.85](http://dx.doi.org/10.1109/ms.2008.85)

547 7.	[Ko AJ, Abraham R, Beckwith L, Blackwell A, Burnett M, Erwig M, et al. The state of the art](http://paperpile.com/b/GCFfyr/pbxy)
548 [in end-user software engineering. ACM Comput Surv. 2011;43: 1–44.](http://paperpile.com/b/GCFfyr/pbxy)
549 8.	[Schulte C, Knobelsdorf M. Attitudes towards computer science-computing experiences as a](http://paperpile.com/b/GCFfyr/guYF)
550 [starting point and barrier to computer science. Proceedings of the third international](http://paperpile.com/b/GCFfyr/guYF)
551 [workshop on Computing education research - ICER ’07. 2007.](http://paperpile.com/b/GCFfyr/guYF)

552	[doi:](http://paperpile.com/b/GCFfyr/guYF)[10.1145/1288580.1288585](http://dx.doi.org/10.1145/1288580.1288585)

553	9.	[Beck L. Remote Sensing and Human Health: New Sensors and New Opportunities.](http://paperpile.com/b/GCFfyr/M19r)

554	[Emerging Infectious Diseases. 2000. pp. 217–227. doi](http://paperpile.com/b/GCFfyr/M19r)[:10.3201/eid0603.000301](http://dx.doi.org/10.3201/eid0603.000301)

555	10. [Fisher P. The pixel: A snare and a delusion. International Journal of Remote Sensing.](http://paperpile.com/b/GCFfyr/Bb34)

556	[1997. pp. 679–685. doi:](http://paperpile.com/b/GCFfyr/Bb34)[10.1080/014311697219015](http://dx.doi.org/10.1080/014311697219015)

557	11. [Jensen J, Narumalani S, Hlady J. Information extraction from remotely sensed data.](http://paperpile.com/b/GCFfyr/oXJC)

558	[Manual of Geospatial Science and Technology. 2001. pp. 298–324.](http://paperpile.com/b/GCFfyr/oXJC)

559	[doi:10.1201/9780203305928.ch19](http://paperpile.com/b/GCFfyr/oXJC)

560 12. [Walter T, Shattuck DW, Baldock R, Bastin ME, Carpenter AE, Duce S, et al. Visualization](http://paperpile.com/b/GCFfyr/Sl4h)
561 [of image data from cells to organisms. Nat Methods. 2010;7: S26–41.](http://paperpile.com/b/GCFfyr/Sl4h)
562 13. [Lee CB. Experience report: CS1 in MATLAB for non-majors, with media computation and](http://paperpile.com/b/GCFfyr/ufYw)
563 [peer instruction. Proceeding of the 44th ACM technical symposium on Computer science](http://paperpile.com/b/GCFfyr/ufYw)
564 [education - SIGCSE ’13. New York, New York, USA: ACM Press; 2013. p. 35.](http://paperpile.com/b/GCFfyr/ufYw)
565 14. [Schadt EE, Linderman MD, Sorenson J, Lee L, Nolan GP. Computational solutions to](http://paperpile.com/b/GCFfyr/hiKM)
566 [large-scale data management and analysis. Nat Rev Genet. 2010;11: 647–657.](http://paperpile.com/b/GCFfyr/hiKM)
567 15. [Christensen K, Rundus D, Fujinoki H, Davis D. A Crash Course for Preparing Students for](http://paperpile.com/b/GCFfyr/x61G)
568 [a First Course in Computing: Did it Work? Journal of Engineering Education. 2002. pp.](http://paperpile.com/b/GCFfyr/x61G)

569	[409–413. doi:10.1002/j.2168-9830.2002.tb00725.x](http://paperpile.com/b/GCFfyr/x61G)

570	16. [Media Computation Teachers Website. [cited 31 Jul 2020]. Available:](http://paperpile.com/b/GCFfyr/M2go)

571	<http://coweb.cc.gatech.edu/mediaComp-teach>

572	17. [Porter L, Guzdial M, McDowell C, Simon B. Success in introductory programming.](http://paperpile.com/b/GCFfyr/0k9Q)

573	[Communications of the ACM. 2013. pp. 34–36. doi:](http://paperpile.com/b/GCFfyr/0k9Q)[10.1145/2492007.2492020](http://dx.doi.org/10.1145/2492007.2492020)

574 18. [Guzdial M, Tew AE. Imagineering inauthentic legitimate peripheral participation: an](http://paperpile.com/b/GCFfyr/AwLH)
575 [instructional design approach for motivating computing education. Proceedings of the 2006](http://paperpile.com/b/GCFfyr/AwLH)
576 [international workshop on Computing education research - ICER ’06. New York, New York,](http://paperpile.com/b/GCFfyr/AwLH)
577 [USA: ACM Press; 2006. p. 51.](http://paperpile.com/b/GCFfyr/AwLH)
578 19. [Blankenship SS, Ruona WEA. Professional Learning Communities and Communities of](http://paperpile.com/b/GCFfyr/waio)
579 [Practice: A Comparison of Models, Literature Review. Online Submission. 2007 [cited 31](http://paperpile.com/b/GCFfyr/waio)
580 [Jul 2020]. Available: http://files.eric.ed.gov/fulltext/ED504776.pdf](http://paperpile.com/b/GCFfyr/waio)
581 20. [Solanki S, McPartlan P, Xu D, Sato BK. Success with EASE: Who benefits from a STEM](http://paperpile.com/b/GCFfyr/hiBD)
582 [learning community? PLoS One. 2019;14: e0213827.](http://paperpile.com/b/GCFfyr/hiBD)
583 21. [Dagley M, Georgiopoulos M, Reece A, Young C. Increasing Retention and Graduation](http://paperpile.com/b/GCFfyr/27Tr)
584 [Rates Through a STEM Learning Community. J Coll Stud Ret. 2016;18: 167–182.](http://paperpile.com/b/GCFfyr/27Tr)
585 22. [Carrino SS, Gerace WJ. Why STEM Learning Communities Work: The Development of](http://paperpile.com/b/GCFfyr/gsd2)
586 [Psychosocial Learning Factors through Social Interaction. Learning Communities:](http://paperpile.com/b/GCFfyr/gsd2)
587 [Research & Practice. 2016;4. Available: http://files.eric.ed.gov/fulltext/EJ1112934.pdf](http://paperpile.com/b/GCFfyr/gsd2)
588 23. [Vogt CM. Faculty as a Critical Juncture in Student Retention and Performance in](http://paperpile.com/b/GCFfyr/tdGl)
589 [Engineering Programs. Journal of Engineering Education. 2008;97: 27–36.](http://paperpile.com/b/GCFfyr/tdGl)
590 24. [Hoadley C. What is a Community of Practice and How Can We Support It? In: Jonassen](http://paperpile.com/b/GCFfyr/52b6)
591 [DH, Land SM, editors. Theoretical foundations of learning environments. Routledge; 2012.](http://paperpile.com/b/GCFfyr/52b6)

592	[pp. 287–300.](http://paperpile.com/b/GCFfyr/52b6)

593	25. [Fencl H, Scheel K. Engaging Students: An Examination of the Effects of Teaching](http://paperpile.com/b/GCFfyr/glYw)

594	[Strategies on Self-Efficacy and Course Climate in a Nonmajors Physics Course. J Coll Sci](http://paperpile.com/b/GCFfyr/glYw)

595	[Teach. 2005;35: 20.](http://paperpile.com/b/GCFfyr/glYw)

596 26. [Beck L, Chizhik A. Cooperative learning instructional methods for CS1: Design,](http://paperpile.com/b/GCFfyr/PKxV)
597 [implementation, and evaluation. ACM Trans Comput Educ. 2013;13: 1–21.](http://paperpile.com/b/GCFfyr/PKxV)
598 27. [Wheeler ER, Wischusen SM. Developing Self-Regulation and Self-Efficacy: A Cognitive](http://paperpile.com/b/GCFfyr/BktK)
599 [Mechanism behind the Success of Biology Boot Camps. Electronic Journal of Science](http://paperpile.com/b/GCFfyr/BktK)
600 [Education. 2014;18. Available: http://files.eric.ed.gov/fulltext/EJ1188281.pdf](http://paperpile.com/b/GCFfyr/BktK)
601 28. [DIVAS Program Resources. [cited 1 Sep 2020]. Available: http://www.doane.edu/divas-](http://paperpile.com/b/GCFfyr/OCmr)
602 [program-resources](http://www.doane.edu/divas-program-resources)
603 29. [Kari L. Jordan, Ben Marwick, Belinda Weaver, Naupaka Zimmerman, Jason Williams,](http://paperpile.com/b/GCFfyr/fb6s)
604 [Tracy Teal, Erin Becker, Jonah Duckles, Beth Duckles, Elizabeth Wickes. Analysis of the](http://paperpile.com/b/GCFfyr/fb6s)
605 [Carpentries’ Long-Term Feedback Survey. Oct 2017 [cited 9 Aug 2020]. Available:](http://paperpile.com/b/GCFfyr/fb6s)
606 [https://carpentries.github.io/assessment/learner-](https://carpentries.github.io/assessment/learner-assessment/archives/2017/code/longtermreport_October2017.html)
607 [assessment/archives/2017/code/longtermreport_October2017.html](https://carpentries.github.io/assessment/learner-assessment/archives/2017/code/longtermreport_October2017.html)
608 30. [Meysenburg M. Image Processing with Python. In: Data Carpentry [Internet]. 14 Mar 2017.](http://paperpile.com/b/GCFfyr/TaWX)
609 [Available: https://datacarpentry.org/image-processing/](http://paperpile.com/b/GCFfyr/TaWX)
610 31. [Doyle E. Python Intro. [cited 20 Jul 2020]. Available: https://eldoyle.github.io/PythonIntro/](http://paperpile.com/b/GCFfyr/BZei)
611 32. [Rosebrook A. Practical Python and OpenCV + Case Studies: An Introductory, Example](http://paperpile.com/b/GCFfyr/6fnX)
612 [Driven Guide to Image Processing and Computer Vision. 2016.](http://paperpile.com/b/GCFfyr/6fnX)
613 33. [Becker E. Image Processing with Python. In: GitHub [Internet]. [cited 1 Sep 2020].](http://paperpile.com/b/GCFfyr/9jH3)
614 [Available:](http://paperpile.com/b/GCFfyr/9jH3) <https://github.com/datacarpentry/image-processing>
615 34. [McDowell C, Werner L, Bullock HE, Fernald J. Pair programming improves student](http://paperpile.com/b/GCFfyr/Nl97)
616 [retention, confidence, and program quality. Commun ACM. 2006;49: 90–95.](http://paperpile.com/b/GCFfyr/Nl97)
617 35. [Williams L, Upchurch RL. In support of student pair-programming. SIGCSE Bull. 2001;33:](http://paperpile.com/b/GCFfyr/Z1g4)

618	[327–331.](http://paperpile.com/b/GCFfyr/Z1g4)

619 36. [Williams L, Wiebe E, Yang K, Ferzli M, Miller C. In Support of Pair Programming in the](http://paperpile.com/b/GCFfyr/wXxD)
620 [Introductory Computer Science Course. null. 2002;12: 197–212.](http://paperpile.com/b/GCFfyr/wXxD)
621 37. [Shell DF, Hazley MP, Soh L, Dee Miller L, Chiriacescu V, Ingraham E. Improving learning](http://paperpile.com/b/GCFfyr/AE4W)
622 [of computational thinking using computational creativity exercises in a college CSI](http://paperpile.com/b/GCFfyr/AE4W)
623 [computer science course for engineers. 2014 IEEE Frontiers in Education Conference (FIE)](http://paperpile.com/b/GCFfyr/AE4W)
624 [Proceedings. 2014. pp. 1–7.](http://paperpile.com/b/GCFfyr/AE4W)
625 38. [Peteranetz MS, Flanigan AE, Shell DF, Soh L-K. Perceived Instrumentality and Career](http://paperpile.com/b/GCFfyr/oDRX)
626 [Aspirations in CS1 Courses: Change and Relationships with Achievement. Proceedings of](http://paperpile.com/b/GCFfyr/oDRX)
627 [the 2016 ACM Conference on International Computing Education Research. New York, NY,](http://paperpile.com/b/GCFfyr/oDRX)
628 [USA: Association for Computing Machinery; 2016. pp. 13–21.](http://paperpile.com/b/GCFfyr/oDRX)
629 39. [Computational thinking for all. [cited 2 Sep 2020]. Available:](http://paperpile.com/b/GCFfyr/FJ1j)
630 <https://www.iste.org/explore/Solutions/Computational-thinking-for-all>
631 40. [Center for Computational Thinking, Carnegie Mellon. [cited 2 Sep 2020]. Available:](http://paperpile.com/b/GCFfyr/gxzC)
632 <https://www.cs.cmu.edu/~CompThink/>
633 41. [Google for Education: Computational Thinking. [cited 2 Sep 2020]. Available:](http://paperpile.com/b/GCFfyr/Nefq)
634 <https://edu.google.com/resources/programs/exploring-computational-thinking/index.html>
635 42. [Wing JM. Computational thinking. Commun ACM. 2006;49: 33–35.](http://paperpile.com/b/GCFfyr/570i)
636 43. [Royce W. Managing the Development of Large Software Systems. Proceedings of IEEE](http://paperpile.com/b/GCFfyr/BSMR)

637	[WESCON. 1970. pp. 328–338.](http://paperpile.com/b/GCFfyr/BSMR)

638 44. [Programming Unplugged: My Robotic Friends. [cited 2 Sep 2020]. Available:](http://paperpile.com/b/GCFfyr/LhSs)
639 <https://curriculum.code.org/csf-1718/courseb/6/>
640 45. [Available N. About IDEA. In: Campus Labs [Internet]. [cited 7 Sep 2020]. Available:](http://paperpile.com/b/GCFfyr/To6r)
641 [https://courseevaluationsupport.campuslabs.com/hc/en-us/articles/360038347493-About-](https://courseevaluationsupport.campuslabs.com/hc/en-us/articles/360038347493-About-IDEA)
642 [IDEA](https://courseevaluationsupport.campuslabs.com/hc/en-us/articles/360038347493-About-IDEA)
643 46. [Nichols M, Cator K, Torres M. Challenge based learner user guide. Redwood City, CA:](http://paperpile.com/b/GCFfyr/ypra)
644 [Digital Promise. 2016; 24–36.](http://paperpile.com/b/GCFfyr/ypra)
645 47. [Ausubel F. C.elegans infection live/dead image set version 1 from the Broad Bioimage](http://paperpile.com/b/GCFfyr/oi2O)
646 [Benchmark Collection. [cited 12 Oct 2020]. Available:](http://paperpile.com/b/GCFfyr/oi2O)
647 <https://bbbc.broadinstitute.org/BBBC010>
648 48. [Michelitsch M, Rössler OE. The “burning ship” and its quasi-Julia sets. Comput Graph.](http://paperpile.com/b/GCFfyr/HvCq)

649	[1992;16: 435–438.](http://paperpile.com/b/GCFfyr/HvCq)

650 49. [Center for Computing in the Liberal Arts. [cited 8 Sep 2020]. Available:](http://paperpile.com/b/GCFfyr/VrlC)
651 <https://www.doane.edu/center-for-computing-in-the-liberal-arts>
652 50. [National Center for Science and Engineering Statistics (NCSES). Women, Minorities, and](http://paperpile.com/b/GCFfyr/Eafi)
653 [Persons with Disabilities in Science and Engineering. National Science Foundation](http://paperpile.com/b/GCFfyr/Eafi)
654 [Alexandria, VA; 2019. Report No.: NSF 19-304.](http://paperpile.com/b/GCFfyr/Eafi)

Figure 1	[Click here to access/download;Figure;file-1.tiff](https://www.editorialmanager.com/pone/download.aspx?id=27681746&guid=45cb7761-0dc7-406e-9880-8997b30ba766&scheme=1)

Figure 2	[Click here to access/download;Figure;file-2.tif](https://www.editorialmanager.com/pone/download.aspx?id=27681748&guid=2b95d00b-6d04-4e48-96a7-fd8cfff31ae6&scheme=1)

Figure 3	[Click here to access/download;Figure;file-3.tif](https://www.editorialmanager.com/pone/download.aspx?id=27681750&guid=169608c2-cb69-4449-bc46-2f13ea74bf2f&scheme=1)

Figure 4	[Click here to access/download;Figure;file-4.tif](https://www.editorialmanager.com/pone/download.aspx?id=27681752&guid=1800ab85-a004-425d-95f7-bb03f0087702&scheme=1)

career_and_efficacy_data

[Click here to access/download](https://www.editorialmanager.com/pone/download.aspx?id=27686519&guid=48146a77-3cab-47b7-b876-a0e09b2fbcad&scheme=1)

# [Supporting Information career_and_efficacy_plosone.xlsx](https://www.editorialmanager.com/pone/download.aspx?id=27686519&guid=48146a77-3cab-47b7-b876-a0e09b2fbcad&scheme=1)

CT_rubric_scorings

[Click here to access/download](https://www.editorialmanager.com/pone/download.aspx?id=27686542&guid=7db026b8-e9d7-41f9-835f-35442d1b1caa&scheme=1)

# [Supporting Information CT_rubric_scorings_plosone.xlsx](https://www.editorialmanager.com/pone/download.aspx?id=27686542&guid=7db026b8-e9d7-41f9-835f-35442d1b1caa&scheme=1)

coding_workshop_data

[Click here to access/download](https://www.editorialmanager.com/pone/download.aspx?id=27686544&guid=3fec00f7-b1f4-417a-b115-8155be5d0f31&scheme=1)

# [Supporting Information coding_workshops_plosone.xlsx](https://www.editorialmanager.com/pone/download.aspx?id=27686544&guid=3fec00f7-b1f4-417a-b115-8155be5d0f31&scheme=1)

[Click here to access/download](https://www.editorialmanager.com/pone/download.aspx?id=27681754&guid=c66d83ec-94a1-4ada-9a58-1ae50a6d77f9&scheme=1)

# [Preprint PDF pone-353987.pdf](https://www.editorialmanager.com/pone/download.aspx?id=27681754&guid=c66d83ec-94a1-4ada-9a58-1ae50a6d77f9&scheme=1)
