# Supplementary material for: Digital imaging and vision analysis in science project improves the self-efficacy and skill of undergraduate students in computational work
Source: PLoS One. 2021 May 5;16(5):e0241946. doi: 10.1371/journal.pone.0241946 (PMC8099079; doi:10.1371/journal.pone.0241946)
Supplement: Attachment — Submitted filename: Response to Reviewers - PLoS 2021 - Resubmission.docx [file pone.0241946.s024.docx]

3/18/2021

Dear Reviewers,

We are pleased to resubmit our manuscript “Digital Imaging and Vision Analysis in Science Project improves the self-efficacy and skill of undergraduate students in computational work”. We thank the reviewers for many helpful suggestions that have greatly improved the manuscript. We have carefully reviewed every comment and have incorporated them into the revised manuscript, which has been submitted with changes tracked.

A summary of these changes include:

- Style changes including the use of ‘Fig’, heading formats, captions for supporting files, and wording choice in select areas
- Additional details regarding participant consent and IRB approval
- Manuscript reorganization including reducing redundancies in the results
- Addition of data comparing the participant demographics with that of the applicant pool
- The addition of analysis of a control group for the purpose of understanding the baseline self-efficacy of participants as well as their retention rates
- Additional information about the recruitment process for the program
- Intervention-specific edits including: softening conclusions in the section on DIVAS Seminar I, additional information about the iterations of DIVAS Seminar II, additional information about other workshop participants and summer research opportunities
- Reporting p-values and effect sizes for all significant results
- Breaking out the career survey into knowledge and interest sub-scores (which is also consistent with previously-published usage of the tools)
- Adding reliability data for the instruments used in the study
- Providing additional information on the development of the computational thinking rubric, including the metric used for scoring interrater reliability, handling and analysis procedures to calculate CT scores including those from pair programming scripts
- Additional analysis and figures including a by-intervention graph for career interest and knowledge and computational thinking across the program
- Addition of IDEA survey data collection and analysis in the methods section
- Cleaning up and adding descriptions to items in supplementary files as needed

Some feedback is more appropriately addressed in this letter. I have detailed them below.

- Overall, we have made a diligent effort to highlight this paper as a PRELIMINARY PILOT study. In that context, we have conducted statistical analyses appropriate to our data set to gather preliminary evidence of potential trends we may want to explore in a large, controlled study. We assert that sharing pilot study data encourages discourse and supports field advancement in a responsible, measured fashion.
- Reviewer 2 has asked the question of whether data from years 1 and 2 for DIVAS Seminar II can be pooled given the changes that were made in the intervention each year. We feel this is appropriate, though we recognize the reviewer’s concern. The same instructor taught the seminar and largely maintained the same overall outcomes and schedule. The changes that were made to the seminar were mostly in the context of the specific applications/problems scholars were presented with, not their overall purpose or context within the course. We have shown the results of each year separately in the manuscript so that readers can weigh that information for themselves.
- Reviewer 1 has asked if a t-test is appropriate given our small sample sizes. We believe it is the most appropriate analysis for our data. However, we have provided all of our data and our analysis methods for a reader to consider in evaluating the findings for themselves.
- Reviewer 2 has raised the concern that our dataset is not usable for answering the questions we have posed. We acknowledge the weakness of the small sample size of our study. However, the measurement of the same subjects over time is a strength of the study. In addition, we have provided an analysis comparing baseline self-efficacy of participants to that of students enrolled in an introductory chemistry course, which are representative of the applicant pool. We have also provided some discussion about how the preliminary findings we are presenting align with previously published results using the same tools.
- Reviewer 1 has asked if we are prone to type I error due to multiple comparisons. We do not feel this applies to our analysis given its scale (no more than six measurements per subject). An ANOVA could be used to better model the repeated measures of each subject, but this analysis is not appropriate given our sample size.
- Reviewer 2 has raised a concern about the 100-point scale used for the self-efficacy instrument. The instrument we have utilized has been designed and validated in published research, as indicated in the manuscript. We are aware that larger scales, such as the one in this instrument, are relatively common in social science research.

Thank you again for your very helpful feedback which has helped us craft a stronger manuscript. We believe this article remains a good fit for PLOS ONE given the potential broad appeal it would generate across educators from a variety of fields. We appreciate the opportunity to submit our revision for your consideration.

Sincerely,

Tessa Durham Brooks
